# Kendrick Analysis and Complex Isotopic Patterns: A Case Study of the Compositional Analysis of Pristine and Heated Polybrominated Flame Retardants by High-Resolution MALDI Mass Spectrometry

**DOI:** 10.5702/massspectrometry.A0079

**Published:** 2020-02-06

**Authors:** Sayaka Nakamura, Hiroaki Sato, Thierry N. J. Fouquet

**Affiliations:** 1 Polymer Chemistry Group, Research Institute for Sustainable Chemistry, National Institute of Advanced Industrial Science and Technology (AIST), Tsukuba, Japan

**Keywords:** Kendrick analysis, polymer, flame retardant, data processing, isotope

## Abstract

The Kendrick analysis is used for the processing and visualization of mass spectra obtained from polymers containing C, H, O and/or Si with simple isotopic patterns (monoisotope=lightest isotope=most intense isotope for short chains). In the case of heteroatoms with complex isotopic patterns, the impact of the chosen isotope on point alignments in Kendrick plots has not been examined extensively. Rich isotopic patterns also make the evaluation of the mass and nature of the repeating unit and end-groups more difficult from the mass spectrum in the case of unknown samples due to the number of peaks and the absence of a monoisotopic peak. Using a polybrominated polycarbonate as running example, we report that horizontal point alignments can be obtained in a Kendrick plot using the mass of the most abundant isotope instead of the monoisotopic mass as is usually done. Rotating the plot (“reverse Kendrick analysis”) helps to accurately evaluate the mass of the most abundant isotope of the repeating unit, as well as the nature of the brominated neutral expelled upon gentle heating (debromination or dehydrobromination). The whole procedure is then applied to the characterization of an unknown polybrominated flame retardant in an industrial formulation before and after heating.

## INTRODUCTION

The Kendrick (mass) analysis has been recently promoted for use in the visualization of mass spectra of polymers.^1)^ It consists in converting the one-dimensional mass spectrum (intensity *vs.* mass-to-charge ratios *m*/*z*) into a two-dimensional map using the fractional parts of *m*/*z* with or without a mass rescaling as a new mathematical dimension along the *y*-axis (with *m*/*z* being the *x*-axis, intensities being retained *via* color coding or point size).^2)^ Mass rescaling (*i.e.*, Kendrick mass) is not mandatory but can be very useful since it lines up the congeners of homopolymeric series horizontally if the corresponding repeating unit (~monomer) is used as the new base unit while all other points are repositioned with other slopes or randomly.^3,4)^ The Kendrick analysis has become a powerful tool for processing and exploring complex mass spectra using commercial, free programs or spreadsheets.

As is implicitly done for all Kendrick analyses reported in the literature, the monoisotopic mass of the repeating unit (noted *R*) is used as the base unit. The monoisotopic mass originates from the most abundant isotopes of the constituent atoms of *R*.^5)^ It corresponds to the lightest isotope for the majority of repeating units that are composed of C, H, O or Si atoms (*e.g.*, ethylene oxide C_2_H_4_O, *m*=44.0262, dimethyl siloxane SiOC_2_H_6_, *m*=74.0188). Heteroatoms with more than one abundant isotope incorporated in the monomer makes the notion of monoisotopic mass, mass spectral analysis and the Kendrick analysis more complex.^6)^ The number of peaks per oligomer is greatly increased, and the mass of the repeating unit itself may not be obviously evaluated from the mass spectrum as is normally done by simply subtracting the *m*/*z* of consecutive oligomers. However, the impact of the isotopic pattern of the repeating unit on Kendrick plots has not been examined in detail.

We report here on several possible issues and solutions related to the Kendrick analysis of polymers with complex isotopic patterns using polybrominated flame retardants as model compounds. We discuss the choice of the isotope for mass rescaling (monoisotopic mass or most abundant isotope) and the nature of its impact on point alignments in a Kendrick plot. The first data processing and compositional analysis of a reference polycarbonate of tetrabromobisphenol A (TBBPA)^7)^ before and after gentle heating was used for the characterization of a pristine and heated industrial formulation of polybutylene terephthalate (PBT) with an unknown polybrominated flame retardant.

## EXPERIMENTAL

The TBBPA-based polycarbonate (noted FRPC, Nihon Kagaku Jouhou, Japan) and a PBT resin containing a brominated flame retardant and antimony trioxide (PBT/FR2300/Sb_2_O_3_ supplied by an undisclosed Japanese company) were dissolved in tetrahydrofuran (denoted as THF) at a concentration of 1 mg mL^−1^. Polymethyl methacrylate standards 1590 and 4000 g mol^−1^ (PMMA, Polymer Laboratories, UK) were dissolved in THF at concentrations of 0.1 and 0.01 mg mL^−1^. *Trans*-2-[3-(4-*tert*-butylphenyl)-2-methyl-2-propenylidene]-malononitrile (TCI, Japan) and sodium trifluoroacetate (NaTFA, Wako, Japan) were dissolved in THF at concentrations of 10 and 1 mg mL^−1^. For the case of heated samples, an FRPC solution was pipetted into a crucible and allowed to air dry or a pellet of a PBT resin was directly placed in another crucible. The crucibles were heated from room temperature to 330°C (FRPC) or 280°C (PBT) at a rate of 100°C/min using an ionRocket device (BioChromato, Japan). Samples were recovered in THF after cooling to room temperature.

Concerning the analyses by mass spectrometry, sample solutions were mixed with DCTB (∼1/10 in volume). The PMMAs were also added to the FRPC solution for internal calibration (∼1/10 in volume). Aliquots of 1 μL of the solutions were deposited on an HST disposable plate (HST Inc., NJ, USA) on top of 1 μL of NaTFA and allowed to air-dry. High-resolution single stage mass spectra were recorded using a JMS-S3000 SpiralTOF mass spectrometer (JEOL, Japan).^8)^

Regarding the data processing, MSTornado control/analysis was used for data acquisition and export while mMass 5.5.0 was used for smoothing, calibration and peak selection.^9,10)^ A compositional analysis using the most abundant isotopes was performed using Mass Mountaineer (RBC software, NH, USA).^11)^ Kendrick plots were computed using our in-house Kendo 1.1 program^12)^ (AIST, Japan) available free of charge for academics. Kendrick masses were calculated using KM=*m*/*z*·*x*/*R* with *m*/*z* the mass-to-charge ratio of an ion, *R* the exact mass of the rescaling unit and *x* the enhancement of resolution (by default, *x*=round(*R*)). Fractional Kendrick masses are calculated using round(KM)-KM.^1)^

## ISSUES AND SOLUTIONS WITH A FLAME-RETARDANT STANDARD

### Rapid evaluation of the repeating unit

**Figure 1:**
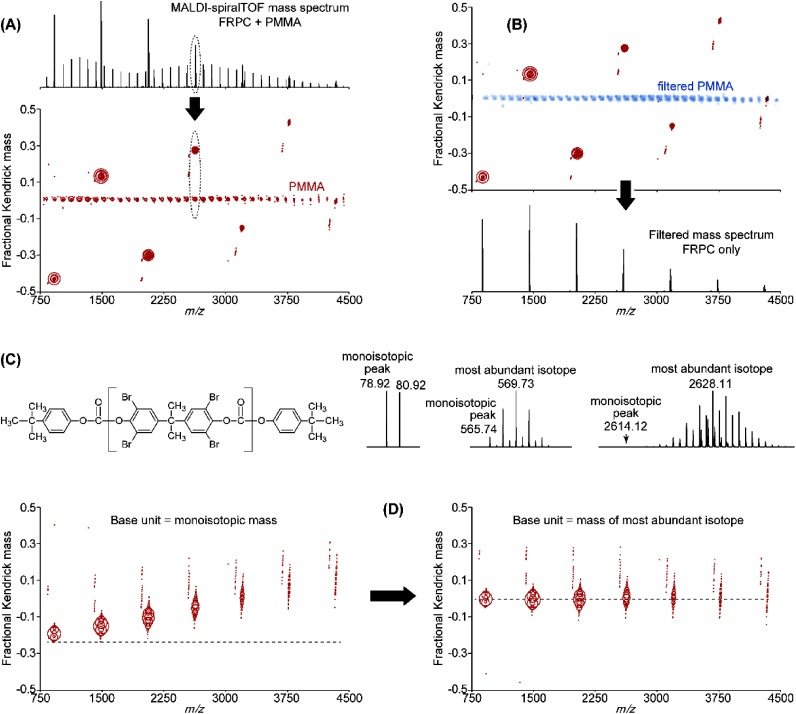
Fig. 1. (A) MALDI mass spectrum of FRPC with PMMA as an internal calibrant and Kendrick plot (base unit: MMA at 100.0524, *x*=100). (B) Filtering of the calibrant using the Kendrick plot. (C) Structure of FRPC and isotopic patterns of bromine, C_16_H_10_O_3_Br_4_, and C_85_H_66_O_15_Br_16_Na. (D) Kendrick plots using the monoisotopic mass of the repeating unit of FRPC at 565.7363 (left, *x*=560) and its most abundant isotope at 569.7324 (right, *x*=564).

The MALDI mass spectrum of FRPC is depicted in Fig. 1A and shows a polymeric series with a large repeating unit spiked with PMMA oligomers for accurate internal calibration. The Kendrick plot greatly improved the visualization of the mass spectral data using methyl methacrylate as the base unit for the mass rescaling (*m*_MMA_ in IUPAC mass scale: 100.0524, set at 100 in the Kendrick scale, enhancement of resolution *x*=100). The PMMA ion series are displayed horizontally at ∼0 since the fractional mass of MMA is null in the new mass scale^13)^ while bromine-containing peaks are distributed along both the *x*- and *y*-axes. The PMMA and FRPC peaks that are overlapped in the mass spectrum are separated thanks to the additional dimension provided by the Kendrick plot (Fig. 1A, dashed lines). The resolution-enhanced Kendrick plot using *x*=99^14)^ permitted two PMMA ion series to be distinguished (Fig. S1) further assigned to (H, H)- and (C_4_H_5_O, H)-ended PMMAs as hypothesized from the simulation of their fractional mass and confirmed from their accurate *m*/*z* ratios (Table S1).

Kendrick plots are also a powerful filtering tool for selecting or erasing features in a congested mass spectrum.^15)^ Using a simple spreadsheet or dedicated software such as Mass Mountaineer or Kendo, PMMA oligomers can be erased from the plot (single selection or multiple zoom in/zoom out and/or different values of *x* to isolate the PMMA oligomers). After filtering, only the brominated components are visible in the Kendrick plot and the associated mass spectrum (Fig. 1B) while mass measurements are still accurate from the internal but invisible calibration.

The structure of FRPC (repeating unit: C_16_H_10_O_3_Br_4_ and end-groups: C_21_H_26_O_3_) is shown in Fig. 1C with the simulated isotopic patterns for bromine and the repeating unit, and the detected isotopic pattern of the sodiated 3-mer in the MALDI-spiralTOF mass spectrum^7)^ (Fig. 1A). Bromine is composed of two stable isotopes ^79^Br and ^81^Br, and the repeating unit *R* contains four bromine atoms. Consequently, the monoisotopic peak is poorly visible in the isotopic pattern of *R* and can no longer be seen in the isotopic pattern of the 3-mer. As a result, all of the detected peaks in the mass spectrum are abundant isotopes different from the monoisotopic masses.^6)^ The monoisotopic mass is always but implicitly used as the rescaling unit for a Kendrick plot^1)^ (*e.g.*, MMA, C_5_H_8_O_2_, 100.0524, Fig. 1A). Doing so for FRPC, the Kendrick plot computed using 565.7363 as the base unit (monoisotopic mass of C_16_H_10_O_3_Br_4_, at 565.7363, set at 566) displays seemingly oblique alignments due to the low relative contribution of the monoisotopic mass to the complex isotopic patterns of the oligomers (Fig. 1D). This pitfall has not yet been reported and should be considered if the polymeric sample of interest is expected to contain heteroatoms with rich isotopic patterns. On the contrary, the use of the mass of the most abundant isotope of C_16_H_10_O_3_Br_4_, at 569.7324 (set at 570 in the new mass scale) generates a main horizontal alignment of the FRPC congeners as expected for a Kendrick analysis.

Considering for a moment that the brominated peaks are unknown, the next steps in a compositional analysis following signal filtering consist of a) determining the mass and elemental composition of the repeating unit and b) evaluating the sum of the masses of the end-groups. The first step is frequently trivial for monomers composed of C, H, O or Si atoms, and is done by subtracting the mass-to-charge ratios of the lightest isotope of two consecutive peaks which provides the monoisotopic mass of *R.* The presence of heteroatoms such as Br makes such a subtraction no longer obvious since it is difficult to choose the peaks corresponding to two consecutive oligomers to be subtracted.

Instead of the monoisotopic mass, subtracting the mass-to-charge ratios of the most intense isotopes of two consecutive oligomers provides the exact mass of the most intense isotope of *R.* The average value found for FRPC is 579.7340 (Table 1). However, numerous programs compute the elemental compositions from an accurate mass by considering only the monoisotopic mass which is useless here. A module has been added recently to Mass Mountaineer that is designed to overcome this issue by computing possible compositions from the mass-measured most abundant isotope^6)^ (Fig. S2). Using the default parameters (DBE=−1 to 100; tolerance: 10 mmu; C=0–50; H=0–100; O=0–10; Br=0–10), five propositions are computed at 569.732361 (C_16_H_10_O_3_Br_4_), 569.740927 (C_18_H_3_O_7_Br_3_), 569.74638 (C_20_H_10_Br_4_), 569.738220 (C_9_H_14_O_8_Br_4_), 569.726134 (C_10_H_19_O_2_Br_5_). Considering their plausibility (C_18_H_3_ is very unlikely), the accuracy (internal calibration with PMMA so low error) and DBE (10, 16, 14, 1 and −1 in that order), the first composition C_16_H_10_O_3_Br_4_ (monoisotopic mass: 565.7363) is the most probable and indeed corresponds to the repeating unit of the FRPC standard^7)^ (Fig. 1A).

**Table 1:** Table 1. Most abundant isotope of the repeating unit for FRPC computed by three methods and examples of accurate mass measurements with assignments of the sum of the masses of the end-groups for the first FRPC congener.

*R* (most abundant isotope)		Assignment	Error (ppm)
Mass difference between consecutive oligomers (maxima of isotopic pattern)	569.7340	569.73236 ↓ C_16_H_10_O_3_Br_4_*	+2.8
Single rotation of Kendrick plot	569.7293*^ or ^**		−5.5
Double rotation of Kendrick plot	569.7414**		+15.8
Example of accurate measurement, 1-mer		Assignment	Error (ppm)
*m*/*z* measured	*m*/*z* theoretical*		
914.9116	914.9143	C_37_H_36_Br_4_O_6_Na^+^ ↓ C_16_H_10_O_3_Br_4_+C_21_H_26_O_3_+Na^+^ (H, O, C and Br isotopes)	−3.0
915.9117	915.9177		−6.6
916.9088	916.9125		−4.0
917.9100	917.9157		−6.3
918.9082	918.9107		−2.8
919.9104	919.9138		−3.7
920.9062	920.9093		−3.3
921.9096	921.9120		−2.6
922.9065	922.9086		−2.3
923.9074	923.9106		−3.5
924.9089	924.9132		−4.7

*using Mass Mountaineer, **using Kendo, first and twelfth horizontal alignments

**Figure 2:**
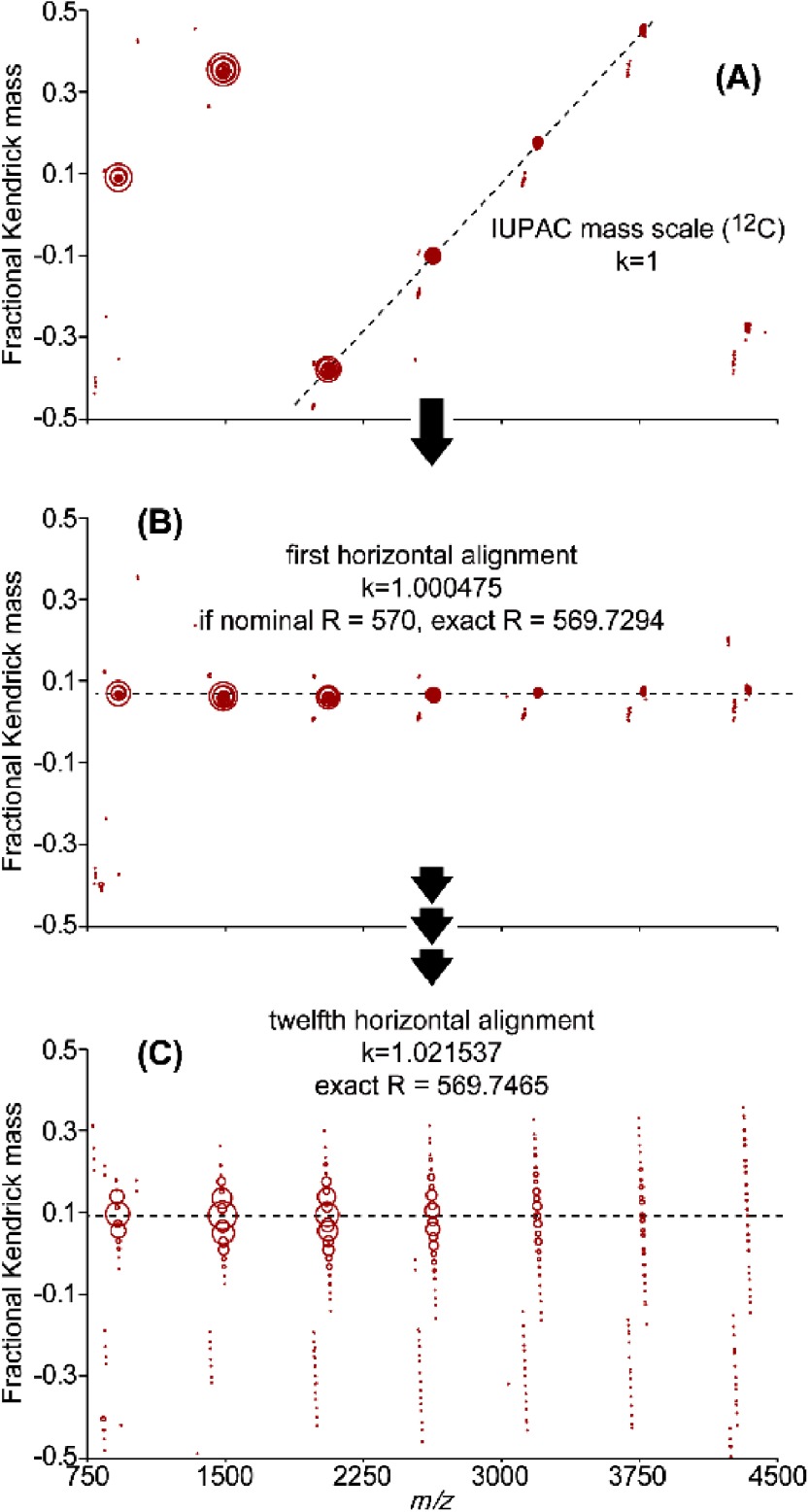
Fig. 2. (A) Kendrick plot from the filtered peak list with brominated species only using ^12^C as the base unit (IUPAC mass scale) as a starting point for the rotation procedure. (B) First horizontal alignment: the mass of *R* can be evaluated with a high degree of accuracy provided its nominal mass is known. (C) Twelfth horizontal alignment with considerable expansion of the isotopic pattern: the mass of *R* is evaluated *ab initio* with a lower accuracy.

The mass of the most intense isotope of *R* can also be evaluated using the “rotating plot” technique.^16)^ Varying the decimal part of a rescaling unit *R* modifies the rescaling ratio *k*=round(*R*)/*R* and the point alignments in a seemingly rotating Kendrick plot. For example, arbitrarily starting from 12, the mass of ^12^C in the IUPAC mass scale, and increasing or decreasing its decimal part to 12.0*xxx* or 11.9*yyy*, there will always be one or several values that permits the targeted point series to be realigned horizontally. The values 12.*xxxx* or 1*y.yyyy* are intimately correlated with the actual value of the repeating unit *R* *via* the rescaling factor *k*=12/12.*xxxx*=11/1*y.yyyy*=round(*R*)/*R.* The Kendrick plot from the filtered peak list of FRPC using ^12^C as the base unit is depicted in Fig. 2A and corresponds to the case of *k*=1. Modifying the value of *k* manually (*e.g.*, with a spreadsheet) or with an appropriate software program (*e.g.*, Mass Mountaineer, Kendo), the Kendrick plot rotates until the point series is clearly aligned horizontally. The rotation can be either clockwise or anticlockwise, and manual or automatic using a 0-sloping procedure at the discretion of the user. An example of a 0-sloping rescaling ratio is *k*=1.00475 obtained using 11.94327 as the base unit (Fig. 2B). If the nominal mass of the most abundant isotope of *R* is known, its accurate mass can be readily computed by dividing the former by *k*. With the nominal *R* at 570, the associated accurate *R* is 570/1.00475∼569.7294 which is in good agreement with the most abundant isotope of C_16_H_10_O_3_Br_4_ at 569.7324 (Table 1).

Alternatively, a “double rotation” procedure provides the exact mass of the most abundant isotope of *R ab initio*.^16)^ Considering the rescaling ratios *k_x_* and *k_y_* found to produce two horizontal alignments of the targeted ion series, the exact mass of *R* is simply *R*=(*y*−*x*)/(*k_y_*−*k_x_*). Consecutive (*y*−*x*=1) or non-consecutive alignments (*y*−*x*>1) can be considered. For example, starting with the first horizontal alignment with 11.94327 as the base unit corresponding to a rescaling ratio *k*_0_=1.000475 (Fig. 2B), the plot can be further rotated until eleven 0 slope cases are reached. Such an extended rotation induces an expansion of the isotopic patterns of the oligomers in the Kendrick plot which further helps at aligning the points.^16)^ At the twelfth 0 slope case (arbitrarily chosen), the base unit is 11.74700 corresponding to a rescaling ratio *k*_12_=1.021537. The exact mass of the most abundant isotope of *R* is computed according to (12−0)/(1.021537−1.000475)=569.7465. This value is still in good agreement with the two previous techniques (Table 1, average values after three “double rotation” procedures). The accuracy is a bit lower (typically ∼0–50 ppm) but this last technique is purely visual and requires no preliminary knowledge. A short video of the single and double rotations using Kendo is uploaded in the Supporting Information. Finally, Mass Mountaineer is used to evaluate the elemental composition associated with the accurate mass of the most abundant isotope, as evaluated by the rotating plot (Table 1).

After assigning the composition of the repeating unit, the final step consists of evaluating the composition of the end-groups. A single stage mass spectrometry analysis can be used to reveal the sum of the masses of the end-groups only, while the individual composition and nature of each of the end-groups can be revealed by tandem mass spectrometry.^17)^ Using the shortest oligomer that can possibly be detected is recommended so as to reduce as much as possible the number of isotopes and take advantage of the highest resolving power of the mass analyzer. Mass Mountaineer is used once more to compute the possible elemental compositions associated with the accurate mass of the most abundant isotope of the 1-mer at *m*/*z* 918.9092 (Fig. S3). Subtracting the elemental composition of the repeating unit, possible elemental compositions for the end-groups and adducted ion can be evaluated in terms of plausibility, accuracy, DBE and the matching of the isotopic pattern simulated by Mass Mountaineer *vs.* the detected pattern. The best match is obtained for C_21_H_26_O_3_Na, in agreement with the *tert*-butylphenyl termination proposed in Fig. 1C and a sodium adduct^7)^ (Table 1).

### Rapid evaluation of the main thermal decomposition pathway

**Figure 3:**
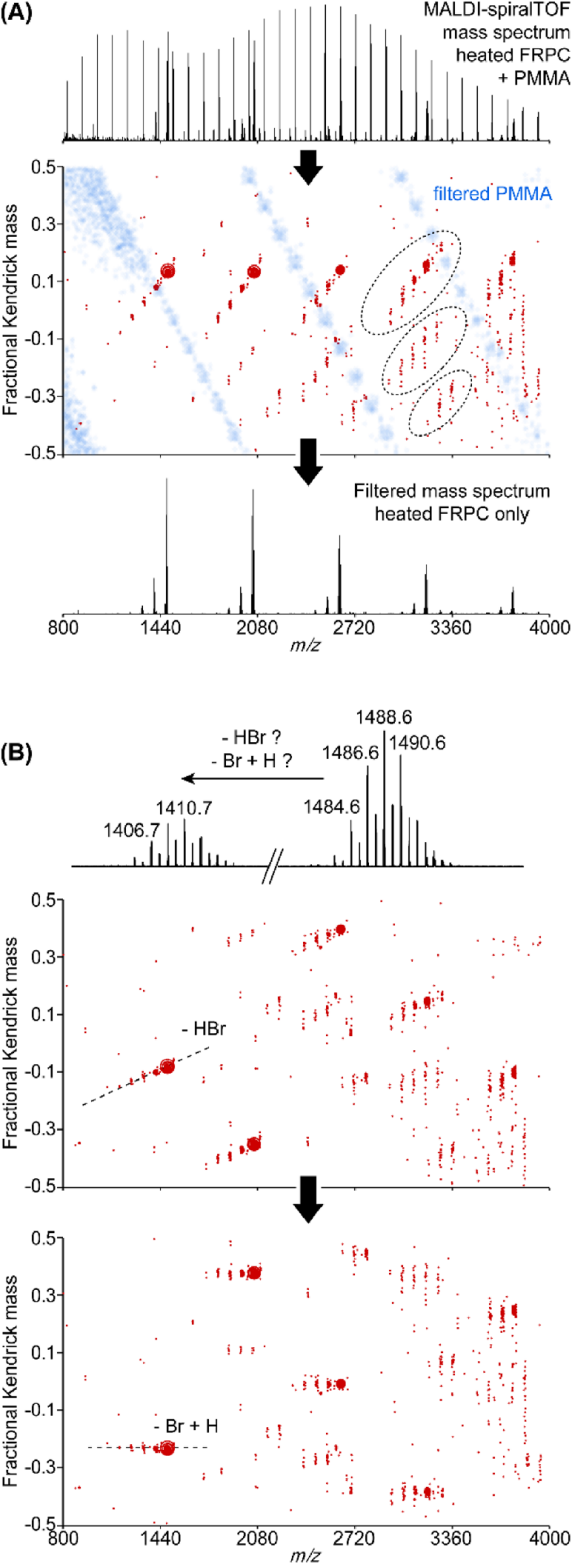
Fig. 3. (A) MALDI mass spectrum of a heated aliquot of FRPC with PMMA as an internal calibrant with its Kendrick plot using the most abundant isotope of C_16_H_10_O_3_Br_4_ as the base unit (*R*=569.7324, *x*=570), and filtered mass spectrum with brominated peaks only. (B) Filtered Kendrick plot using HBr as the base unit (*R*=79.9262, *x*=80) or −Br+H as the base unit (*R*=77.9105, *x*=78).

The MALDI mass spectrum of a heated FRPC sample spiked with PMMA is shown in Fig. 3A. The Kendrick plot computed with MMA as the base unit (Fig. S3) was used to filter the PMMA signal to process only peaks that contain bromine. Adopting the mass of the most abundant isotope of the computed repeating unit C_16_H_10_O_3_ as the base unit (*R*=569.7324, *x*=570), the associated Kendrick plot displays several point series with horizontal and oblique alignments. Accurate mass measurements and assignments of the three peak series aligned horizontally are listed in Table S2, the major series being intact FRPC while the two minor series denoted by circled with dotted lines are hypothetically assigned to branched structures formed upon the loss of bromine radicals and recombination (Fig. S3). Assignments were conducted using Mass Mountaineer to compute possible elemental compositions from the masses of the most abundant isotopes of the congeners.

The supernumerary series that are aligned obliquely in the Kendrick plot from every congener of the main and minor distributions appears to arise from the release of a neutral species upon heating. Peaks are forming another “polymeric distribution” with the expelled neutral species being equivalent to a repeating unit. A Kendrick analysis may be of help in finding this repetitive neutral loss using either the rotating plot if the nature of the neutral species is fully unknown (*vide supra*) or simply by evaluating the slope of the point alignments if neutral species are hypothesized. Possible moieties eliminated upon the heating of FRPC may be either radical Br· (replaced by H·, debromination) or HBr (dehydrobromination).^18)^ Evaluating such a neutral loss from the mass spectrum is not obvious, since a +/− 2 Da variation (−79.9 or −77.9 Da) is embedded in the complex isotopic patterns of the brominated congeners (Fig. 3B). If the eliminated moiety is correctly evaluated, the series aligned obliquely in Fig. 3A will realign horizontally in the corresponding Kendrick plot using the expelled neutral species as the base unit. Figure 3B shows the filtered Kendrick plot using HBr as the base unit (*R*=79.9262, *x*=80). The sub-series line up obliquely, demonstrating that the eliminated moiety is *not* HBr. On the other hand, setting −Br+H as the base unit (*R*=77.9105, *x*=78) generates horizontal alignments which support the hypothesis that debromination is the major pathway for FRPC. This elimination deduced within a minute from the Kendrick plots is later confirmed using the accurate mass measurements of the shortest congeners with a lengthy comparison of experimental and simulated values (Table S2). Of note, the mass measurements tend to be less and less accurate with increasing size of the oligomers which makes the use of *m*/*z* values somewhat unreliable, in opposition to the graphical analysis using a Kendrick plot.

## APPLICATION TO PRISTINE AND HEATED INDUSTRIAL FORMULATION

**Figure 4:**
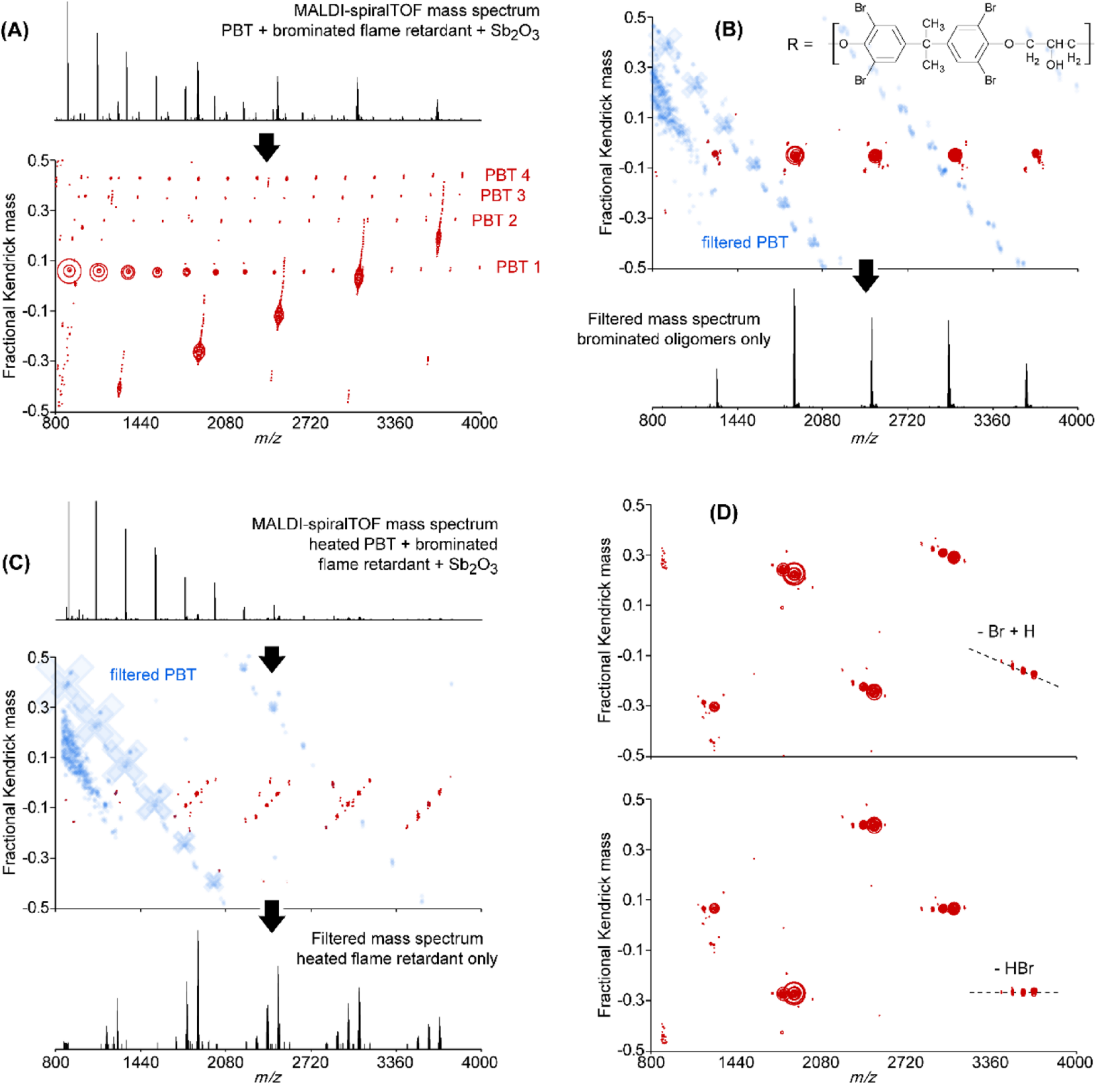
Fig. 4. (A) Top: MALDI mass spectrum of a commercially available PBT formulation. Bottom: Kendrick plot using BT as the base unit (*R*=220.0763, *x*=219). (B) Top: Filtered Kendrick plot using the most abundant isotope of the repeating unit C_18_H_16_O_3_Br_4_ evaluated using a rotating plot as the base unit (*R*=599.7793, *x*=600). Bottom: filtered mass spectrum with brominated peaks only. (C) Filtering of PBT peaks from the MALDI mass spectrum of the heated PBT using a Kendrick plot. (D) Filtered Kendrick plot using −Br+H (*R*=77.9105, *x*=78) or −HBr as the base unit (*R*=79.9262, *x*=80).

Based on the procedure developed with the FRPC standard, an example of an application is proposed for an industrial material labelled as “PBT+FR2300+Sb_2_O_3_” known to contain a brominated flame retardant with no other details. Its MALDI mass spectrum (external calibration, PMMA) and the associated resolution-enhanced Kendrick plot (BT at 220.0763 as the rescaling unit, *x*=219) are shown in Fig. 4A. Four series of PBT are clearly observed *via* their horizontal point alignments, later assigned to cyclic chains (PBT 1), C_14_H_12_O_5_-ended chains (PBT 2), C_4_H_8_O-ended chains (PBT 3) and C_4_H_10_O_2_-ended chains (PBT 4). Accurate mass measurements are listed in Table S3. Cyclic chains are common by-products that are found in the low mass range of industrial polymers that are formed upon polycondensation and can be advantageously used as an internal calibrant in the replacement of PMMA. All of the PBT-related peaks are nonetheless erased in the Kendrick plots and only the polybrominated peaks of interest are revealed.

The accurate mass of the most abundant isotope of the repeating unit of the brominated polymer is evaluated as before by: a) the difference in the mass of the most abundant isotopes of two consecutive oligomers in the mass spectrum; b) a single rotating plot using an estimated nominal mass and c) a double rotating plot (Figs. S4 and S5). The results are listed in Table 2 with an average mass at 599.79 further assigned to C_18_H_16_O_3_Br_4_ using Mass Mountaineer. A possible associated structure could be a polymer of tetrabromobisphenol A diglycidyl ether (ring-opening of epoxy) as depicted in Fig. 4B (proposed CAS 68928-70-1). Using its most abundant isotope as the rescaling unit for the Kendrick plot, neat horizontal alignments are reached for several series isolated one by one by consecutive selections in the plot and assigned to hydrated series (+H_2_O, +2H_2_O from the mains series) and a −C_3_H_4_O defect (Fig. S6). The three minor series are consistent with a diglycidicyl ether repeating unit/end-groups for the main series (−C_3_H_4_O=defective chain lacking one glycidyl ether moiety; +H_2_O/+2H_2_O=hydration of epoxy groups due to prolonged storage).

**Table 2:** Table 2. Most abundant isotope of the repeating unit for the brominated flame retardant in PBT computed by three different methods and examples of accurate mass measurements with assignment of the sum of the masses of the end-groups for the first congener.

*R* (most abundant isotope)		Assignment	Error (ppm)
Mass difference between consecutive oligomers (maxima of isotopic pattern)	599.7799	599.77934 ↓ C_18_H_16_O_3_Br_4_*	+0.9
Single rotation of Kendrick plot	599.7800*^ or ^**		+1.1
Double rotation of Kendrick plot	599.8020**		+37.8
Example of accurate measurement, 1-mer		Assignment	Error (ppm)
*m*/*z* measured	*m*/*z* theoretical*		
1270.5858	1270.5826	C_39_H_36_Br_8_O_7_Na^+^ ↓ C_18_H_16_O_3_Br_4_+C_21_H_20_O_4_Br_4_+Na^+^ (H, O, C and Br isotopes)	2.5
N.D.	1271.5860		N.D.
1272.5777	1272.5806		−2.3
1273.5763	1273.5839		−6.0
1274.5753	1274.5787		−2.7
1275.5788	1275.5820		−2.5
1276.5744	1276.5769		−1.9
1277.5746	1277.5800		−4.2
1278.5710	1278.5751		−3.2
1279.5762	1279.5780		−1.5
1280.5692	1280.5734		−3.3
1281.5709	1281.5762		−4.1
1282.5690	1282.5719		−2.3
1283.5715	1283.5744		−2.2
1284.5661	1284.5707		−3.6
1285.5694	1285.5728		−2.6
1286.5607	1286.5706		−7.7

*using Mass Mountaineer, **using Kendo, first and thirteenth horizontal alignments

As a final step, the nature of the brominated neutral component that is expelled under gentle heating was also examined for this PBT resin. The Kendrick plot using the most abundant isotope of C_18_H_16_O_3_Br_4_ as the base unit from the MALDI mass spectrum of heated PBT (Fig. S7) is used to filter only the brominated peaks (Fig. 4C). The filtered peak list without PBT or a background signal is finally replotted using −Br+H (*R*=77.9105, *x*=78) and −HBr (*R*=79.9262, *x*=80) as base units (Fig. 4D). Horizontal alignments are obtained for HBr, instantly indicating that dehydrobromination is the major pathway for this industrial formulation, unlike the findings for FRPC (Table S4).

## CONCLUSION

In the case of heteroatoms with a complex isotopic pattern incorporated into a repeating unit, using the mass of the most abundant isotope of the repeating unit for the computation of Kendrick masses to obtain favorable horizontal alignments in Kendrick plots is highly recommended. In the case of unknown samples, rotating the Kendrick plot provides the accurate mass of the most abundant isotope of the repeating unit, from which its elemental composition can be computed using appropriate software programs. The Kendrick analysis is sufficiently powerful to filter out background signals and isolate minor but important features from standard or industrial formulations. The technique can be used for any heteroatom with complex isotopic patterns beyond bromine.

## Notes

Mass Spectrom (Tokyo) 2020; 9(1): A0079
