# Supplementary material for: Kendrick Analysis and Complex Isotopic Patterns: A Case Study of the Compositional Analysis of Pristine and Heated Polybrominated Flame Retardants by High-Resolution MALDI Mass Spectrometry
Source: Mass Spectrom (Tokyo). 2020 Feb 6;9(1):A0079. doi: 10.5702/massspectrometry.A0079 (PMC7039712; doi:10.5702/massspectrometry.A0079)
Supplement: Supplementary Data [file massspectrometry-9-1-A0079-s001.pdf]

Kendrick analysis and complex isotopic patterns: a case study of the compositional analysis of pristine and heated polybrominated flame retardants by high-resolution MALDI mass spectrometry

Sayaka Nakamura, Hiroaki Sato, Thierry N. J. Fouquet

Polymer Chemistry Group, Research Institute for Sustainable Chemistry, National Institute of Advanced Industrial Science and Technology (AIST), Tsukuba, Japan.

* to whom correspondence should be addressed: thierry.fouquet@aist.go.jp

Supporting Information

Content

Figure S1. Kendrick plot from the MALDI-spiralTOF mass spectrum of FRPC. 2

Tables S1. Accurate mass measurements and assignments for the PMMA and FRPC main series. 2

Figure S2. Screenshots of Mass Mountaineer 4

Supporting File 1. Short video about the rotating plot 6

Figure S3. Top: MALDI-spiralTOF mass spectrum of heated FRPC (T=330 degC, 5 min) with PMMA as internal calibrant. Bottom: Kendrick plot using R=100.0524 and x=100. **Scheme S1**. Hypothetical structures of the two minor series detected in the mass spectrum of the heated FRPC sample. 6

Tables S2. Accurate mass measurements and assignments for the PMMA and FRPC main series. Errors are expressed in ppm. Peaks are selected from the Kendrick plots. PMMA peaks are omitted (same series as in the previous table). 7

Figure S4. (A) Kendrick plot from the filtered peak list of PBT / brominated flame retardant / Sb_2O_3 with brominated species only using ^{12}C as the base unit (IUPAC mass scale) as a starting point for the rotation procedure. (B) First horizontal alignment: the mass of R can be evaluated with a high degree of accuracy if its nominal mass is known. (C) Thirteenth horizontal alignment with considerable expansion of the isotopic pattern: the mass of R is evaluated ab initio with lower accuracy. 14

Figure S5. Screenshots of Mass Mountaineer 14

Figure S6. Kendrick plot from the filtered peak list of PBT resin using XXX as the base unit. Structures of the minor series. 16

Table S3. Accurate mass measurements and assignments for the main series (PBT and brominated flamed retardant) detected in the PBT / flame retardant / Sb2O3 sample. 16

Figure S7. MALDI-spiralTOF mass spectrum of heated PBT / brominated flame retardant / Sb_2O_3 21

Table S4. Accurate mass measurements and assignments for the loss of bromine (-Br + H or -HBr) from the main series detected in the PBT / flame retardant / Sb2O3 sample after heating 22

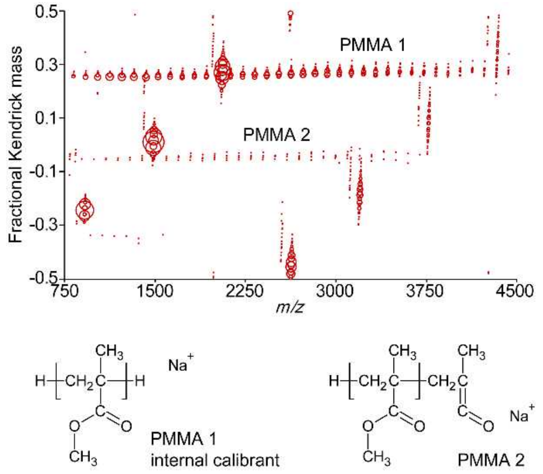

Figure S1. Kendrick plot from the MALDI-spiralTOF mass spectrum of brominated poly(carbonate) FRPC used as a reference compound with PMMA as internal calibrant with R=100.0524 and x=99. Structures of the two PMMA series.

Tables S1. Accurate mass measurements and assignments for the PMMA and FRPC main series. Errors are expressed in ppm. Peaks are selected from Kendrick plots using kendo (polygon selection / export). Errors are indifferently computed using Excel from the peak list or kendo from the Kendrick plot (simulation + polygon selection / compute / error / plot / export).

sodiated PMMA 1, used as internal calibrant

$m/z_{measured}$	$m/z_{theoretical}$	error	assignment
825.4207	-	-	$[H_2(C_5H_8O_2)_8 + Na]^+$
925.4729	-	-	$[H_2(C_5H_8O_2)_9 + Na]^+$
1025.5299	-	-	$[H_2(C_5H_8O_2)_{10} + Na]^+$
1125.5807	-	-	$[H_2(C_5H_8O_2)_{11} + Na]^+$
1225.6342	-	-	$[H_2(C_5H_8O_2)_{12} + Na]^+$
1325.6877	-	-	$[H_2(C_5H_8O_2)_{13} + Na]^+$
1425.7413	-	-	$[H_2(C_5H_8O_2)_{14} + Na]^+$
1525.7929	-	-	$[H_2(C_5H_8O_2)_{15} + Na]^+$
1625.8452	-	-	$[H_2(C_5H_8O_2)_{16} + Na]^+$
1725.9010	-	-	$[H_2(C_5H_8O_2)_{17} + Na]^+$
1825.9509	-	-	$[H_2(C_5H_8O_2)_{18} + Na]^+$
1926.0034	-	-	$[H_2(C_5H_8O_2)_{19} + Na]^+$
2026.0537	-	-	$[H_2(C_5H_8O_2)_{20} + Na]^+$
2126.1089	-	-	$[H_2(C_5H_8O_2)_{21} + Na]^+$
2226.1621	-	-	$[H_2(C_5H_8O_2)_{22} + Na]^+$
2326.2155	-	-	$[H_2(C_5H_8O_2)_{23} + Na]^+$
2526.3168	-	-	$[H_2(C_5H_8O_2)_{25} + Na]^+$
2726.4222	-	-	$[H_2(C_5H_8O_2)_{27} + Na]^+$
2826.4714	-	-	$[H_2(C_5H_8O_2)_{28} + Na]^+$
2926.5288	-	-	$[H_2(C_5H_8O_2)_{29} + Na]^+$
3026.5776	-	-	$[H_2(C_5H_8O_2)_{30} + Na]^+$
3126.6269	-	-	$[H_2(C_5H_8O_2)_{31} + Na]^+$
3226.6805	-	-	$[H_2(C_5H_8O_2)_{32} + Na]^+$
3326.7353	-	-	$[H_2(C_5H_8O_2)_{33} + Na]^+$
3426.7873	-	-	$[H_2(C_5H_8O_2)_{34} + Na]^+$
3526.8440	-	-	$[H_2(C_5H_8O_2)_{35} + Na]^+$

sodiated PMMA 2

$m/z_{measured}$	$m/z_{theoretical}$	error	assignment
893.4475	893.4511	-4.0	$[C_4H_6O(C_5H_8O_2)_8 + Na]^+$
993.5010	993.5035	-2.5	$[C_4H_6O\ (C_5H_8O_2)_9 + Na]^+$
1093.5556	1093.5559	-0.3	$[C_4H_6O\ (C_5H_8O_2)_{10} + Na]^+$
1193.6102	1193.6084	1.5	$[C_4H_6O\ (C_5H_8O_2)_{11} + Na]^+$
1293.6617	1293.6608	0.7	$[C_4H_6O\ (C_5H_8O_2)_{12} + Na]^+$
1393.7122	1393.7132	-0.7	$[C_4H_6O\ (C_5H_8O_2)_{13} + Na]^+$
1493.7653	1493.7656	-0.2	$[C_4H_6O\ (C_5H_8O_2)_{14} + Na]^+$
1593.8223	1593.8181	2.6	$[C_4H_6O\ (C_5H_8O_2)_{15} + Na]^+$
1693.8741	1693.8705	2.1	$[C_4H_6O\ (C_5H_8O_2)_{16} + Na]^+$
1793.9264	1793.9229	1.9	$[C_4H_6O\ (C_5H_8O_2)_{17} + Na]^+$
1893.9813	1893.9754	3.1	$[C_4H_6O\ (C_5H_8O_2)_{18} + Na]^+$
1994.0298	1994.0278	1.0	$[C_4H_6O\ (C_5H_8O_2)_{19} + Na]^+$
2094.0858	2094.0802	2.7	$[C_4H_6O\ (C_5H_8O_2)_{20} + Na]^+$
2194.1358	2194.1327	1.4	$[C_4H_6O\ (C_5H_8O_2)_{21} + Na]^+$
2294.1864	2294.1851	0.6	$[C_4H_6O\ (C_5H_8O_2)_{22} + Na]^+$
2394.2355	2394.2375	-0.9	$[C_4H_6O\ (C_5H_8O_2)_{23} + Na]^+$
2494.2853	2494.2899	-1.8	$[C_4H_6O\ (C_5H_8O_2)_{24} + Na]^+$
2594.3354	2594.3424	-2.7	$[C_4H_6O\ (C_5H_8O_2)_{25} + Na]^+$
2694.3976	2694.3948	1.1	$[C_4H_6O\ (C_5H_8O_2)_{26} + Na]^+$
2794.4473	2794.4472	0.0	$[C_4H_6O\ (C_5H_8O_2)_{27} + Na]^+$
2894.4802	2894.4997	-6.7	$[C_4H_6O\ (C_5H_8O_2)_{28} + Na]^+$

Sodiated main FRPC series

m/z$_{measured}$	*m/z*$_{theoretical}$	error	assignment
914.9116	914.9143	-3.0	$[C_{16}H_{10}O_3Br_4(C_{21}H_{26}O_3) + Na]^+$
915.9117	915.9177	-6.6	
916.9088	916.9125	-4.0	
917.9100	917.9157	-6.3	
918.9082	918.9107	-2.8	
919.9104	919.9138	-3.7	
920.9062	920.9093	-3.3	
921.9096	921.9120	-2.6	
922.9065	922.9086	-2.3	
923.9074	923.9106	-3.5	
1482.6534	1482.6488	3.1	$[C_{16}H_{10}O_3Br_4(C_{21}H_{26}O_3)_2 + Na]^+$
1483.6558	1483.6521	2.5	
1484.6497	1484.6470	1.8	
1485.6528	1485.6501	1.8	
1486.6473	1486.6453	1.4	
1487.6511	1487.6482	1.9	
1488.6463	1488.6436	1.8	
1489.6507	1489.6463	2.9	
1490.6466	1490.6422	3.0	
1491.6472	1491.6445	1.8	
1492.6438	1492.6409	1.9	
1493.6451	1493.6429	1.5	
1494.6423	1494.6402	1.4	
1495.6443	1495.6416	1.8	
1496.6466	1496.6404	4.2	
2050.3877	2050.3834	2.1	$[C_{16}H_{10}O_3Br_4(C_{21}H_{26}O_3)_3 + Na]^+$
2051.3891	2051.3865	1.3	
2052.3804	2052.3816	-0.6	
2053.3875	2053.3845	1.4	
2054.3844	2054.3799	2.2	
2055.3868	2055.3826	2.0	
2056.3790	2056.3783	0.4	
2057.3819	2057.3807	0.6	
2058.3798	2058.3767	1.5	
2059.3832	2059.3789	2.1	
2060.3763	2060.3752	0.5	
2061.3802	2061.3771	1.5	
2062.3738	2062.3739	0.0	
2063.3781	2063.3755	1.3	
2064.3723	2064.3727	-0.2	
2065.3771	2065.3739	1.5	
2066.3717	2066.3719	-0.1	
2067.3770	2067.3727	2.1	
2068.3721	2068.3715	0.3	
2618.1126	2618.1180	-2.1	$[C_{16}H_{10}O_3Br_4(C_{21}H_{26}O_3)_4 + Na]^+$
2619.1145	2619.1209	-2.4	
2620.1107	2620.1163	-2.1	
2621.1130	2621.1190	-2.3	
2622.1096	2622.1146	-1.9	
2623.1123	2623.1171	-1.8	
2624.1093	2624.1129	-1.4	
2625.1123	2625.1152	-1.1	
2626.1097	2626.1114	-0.6	
2627.1072	2627.1134	-2.3	
2628.1049	2628.1098	-1.9	
2629.1029	2629.1116	-3.3	
2630.1010	2630.1083	-2.8	
2631.1052	2631.1098	-1.8	
2632.1037	2632.1069	-1.3	
2633.1023	2633.1081	-2.2	
2634.1012	2634.1057	-1.7	
2635.1002	2635.1066	-2.4	
2636.0995	2636.1045	-1.9	
2637.0989	2637.1051	-2.4	
2638.1045	2638.1036	0.3	
2639.0984	2639.1039	-2.1	

m/z$_{measured}$	*m/z*$_{theoretical}$	error	assignment
3187.8328	3187.8510	-5.7	$[C_{16}H_{10}O_3Br_4(C_{21}H_{26}O_3)_5 + Na]^+$
3188.8473	3188.8534	-1.9	
3189.8359	3189.8493	-4.2	
3190.8377	3190.8516	-4.3	
3191.8332	3191.8477	-4.5	
3192.8353	3192.8497	-4.5	
3193.8311	3193.8461	-4.7	
3194.8335	3194.8479	-4.5	
3195.8296	3195.8445	-4.7	
3196.8323	3196.8461	-4.3	
3197.8287	3197.8430	-4.5	
3198.8318	3198.8443	-3.9	
3199.8285	3199.8415	-4.1	
3200.8254	3200.8426	-5.4	
3201.8289	3201.8401	-3.5	
3202.8261	3202.8409	-4.6	
3203.8299	3203.8387	-2.7	
3204.8274	3204.8393	-3.7	
3205.8250	3205.8374	-3.9	
3206.8294	3206.8378	-2.6	
3207.8273	3207.8363	-2.8	
3208.8254	3208.8364	-3.4	
3758.5600	3758.5861	-6.9	$[C_{16}H_{10}O_3Br_4(C_{21}H_{26}O_3)_6 + Na]^+$
3759.5627	3759.5825	-5.2	
3760.5585	3760.5842	-6.8	
3761.5615	3761.5809	-5.1	
3762.5717	3762.5824	-2.8	
3763.5608	3763.5793	-4.9	
3764.5571	3764.5806	-6.2	
3765.5606	3765.5777	-4.5	
3766.5572	3766.5788	-5.7	
3767.5539	3767.5762	-5.9	
3768.5649	3768.5771	-3.2	
3769.5548	3769.5747	-5.3	
3770.5519	3770.5754	-6.2	
3771.5491	3771.5732	-6.4	
3772.5606	3772.5737	-3.5	
3773.5510	3773.5718	-5.5	
3774.5416	3774.5721	-8.1	
3775.5606	3775.5704	-2.6	
3776.5585	3776.5706	-3.2	
3777.5565	3777.5691	-3.3	
3778.5547	3778.5691	-3.8	
4327.3008	4327.3173	-3.8	$[C_{16}H_{10}O_3Br_4(C_{21}H_{26}O_3)_7 + Na]^+$
4328.2706	4328.3188	-11.1	
4329.2860	4329.3157	-6.9	
4330.2863	4330.3169	-7.1	
4331.2792	4331.3141	-8.0	
4332.3101	4332.3151	-1.2	
4333.3033	4333.3125	-2.1	
4334.2813	4334.3134	-7.4	
4335.2823	4335.3109	-6.6	
4336.2757	4336.3116	-8.3	
4337.2845	4337.3093	-5.7	
4338.2858	4338.3099	-5.6	
4339.2796	4339.3078	-6.5	
4340.2811	4340.3082	-6.3	
4341.2827	4341.3063	-5.4	
4342.2844	4342.3066	-5.1	
4343.2711	4343.3049	-7.8	
4344.2807	4344.3050	-5.6	
4345.2904	4345.3035	-3.0	
4346.2774	4346.3034	-6.0	
4347.2873	4347.3021	-3.4	
4348.2593	4348.3019	-9.8	

Loss of bromine from the sodiated main series (-HBr or -Br + H)

- Br + H or – HBr from main series						
$m/z_{measured}$	$m/z_{theoretical}$	error	assignment	$m/z_{theoretical}$	error	assignment
1976.4696	1976.4695	0.0	$[C_{16}H_{11}O_3Br_3(C_{21}H_{26}O_3)_3 + Na]^+$ = - Br + H	1976.4523	8.8	$[C_{16}H_9O_3Br_3(C_{21}H_{26}O_3)_3 + Na]^+$ = - HBr
1977.4682	1977.4722	-2.0		1977.4547	6.8	
1978.4670	1978.4679	-0.5		1978.4508	8.2	
1979.4712	1979.4703	0.4		1979.4529	9.3	
1980.4705	1980.4664	2.1		1980.4494	10.6	
1981.4701	1981.4685	0.8		1981.4512	9.6	
1982.4648	1982.4651	-0.1		1982.4483	8.3	
1983.4649	1983.4668	-1.0		1983.4496	7.7	
2546.1998	2546.2026	-1.1	$[C_{16}H_{11}O_3Br_3(C_{21}H_{26}O_3)_4 + Na]^+$ = - Br + H	2546.1854	5.6	$[C_{16}H_9O_3Br_3(C_{21}H_{26}O_3)_4 + Na]^+$ = - HBr
2547.1994	2547.2048	-2.1		2547.1873	4.8	
2548.1935	2548.2010	-3.0		2548.1839	3.8	
2549.1994	2549.2030	-1.4		2549.1855	5.4	
2550.1997	2550.1996	0.0		2550.1825	6.7	
2551.2001	2551.2012	-0.4		2551.1839	6.4	
2552.1950	2552.1982	-1.3		2552.1812	5.4	
2553.1900	2553.1995	-3.7		2553.1823	3.0	
2554.1852	2554.1969	-4.6		2554.1801	2.0	
3115.9264	3115.9358	-3.0	$[C_{16}H_{11}O_3Br_3(C_{21}H_{26}O_3)_5 + Na]^+$ = - Br + H	3115.9186	2.5	$[C_{16}H_9O_3Br_3(C_{21}H_{26}O_3)_5 + Na]^+$ = - HBr
3116.9294	3116.9374	-2.6		3116.9200	3.0	
3117.9197	3117.9342	-4.7		3117.9171	0.8	
3118.9166	3118.9357	-6.1		3118.9183	-0.5	
3119.9201	3119.9327	-4.1		3119.9157	1.4	
3120.9237	3120.9339	-3.3		3120.9166	2.3	
3121.9082	3121.9313	-7.4		3121.9143	-2.0	
3122.9186	3122.9323	-4.4		3122.9150	1.2	
3123.9098	3123.9300	-6.4		3123.9130	-1.0	
3685.6396	3685.6690	-8.0	$[C_{16}H_{11}O_3Br_3(C_{21}H_{26}O_3)_6 + Na]^+$ = - Br + H	3685.6518	-3.3	$[C_{16}H_9O_3Br_3(C_{21}H_{26}O_3)_6 + Na]^+$ = - HBr
3686.6326	3686.6702	-10.2		3686.6528	-5.5	
3687.6466	3687.6674	-5.6		3687.6503	-1.0	
3689.6331	3689.6659	-8.9		3689.6488	-4.2	
3690.6336	3690.6667	-9.0		3690.6494	-4.3	
3691.6412	3691.6645	-6.3		3691.6474	-1.7	

Figure S2. screenshots of Mass Mountaineer for the computation of elemental compositions from the most abundant isotope

a) repeating unit

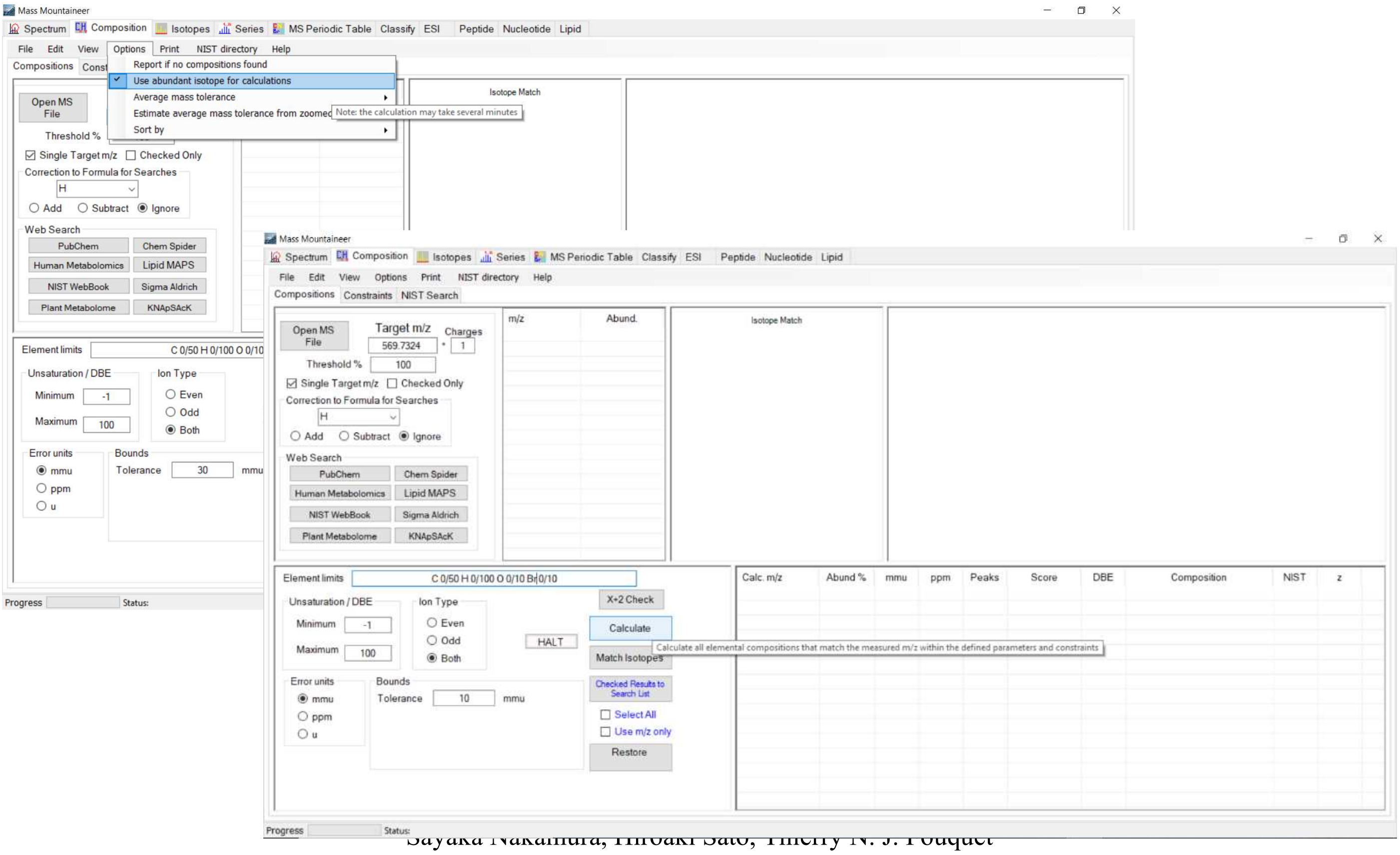

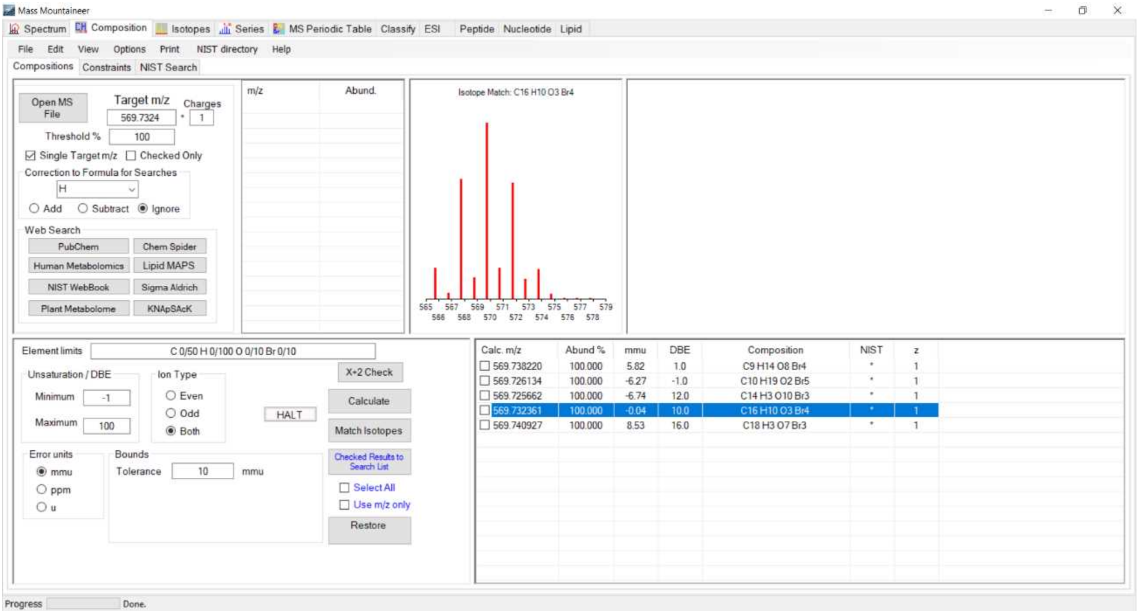

b) first oligomer (wrong and correct composition based on the isotopic pattern)

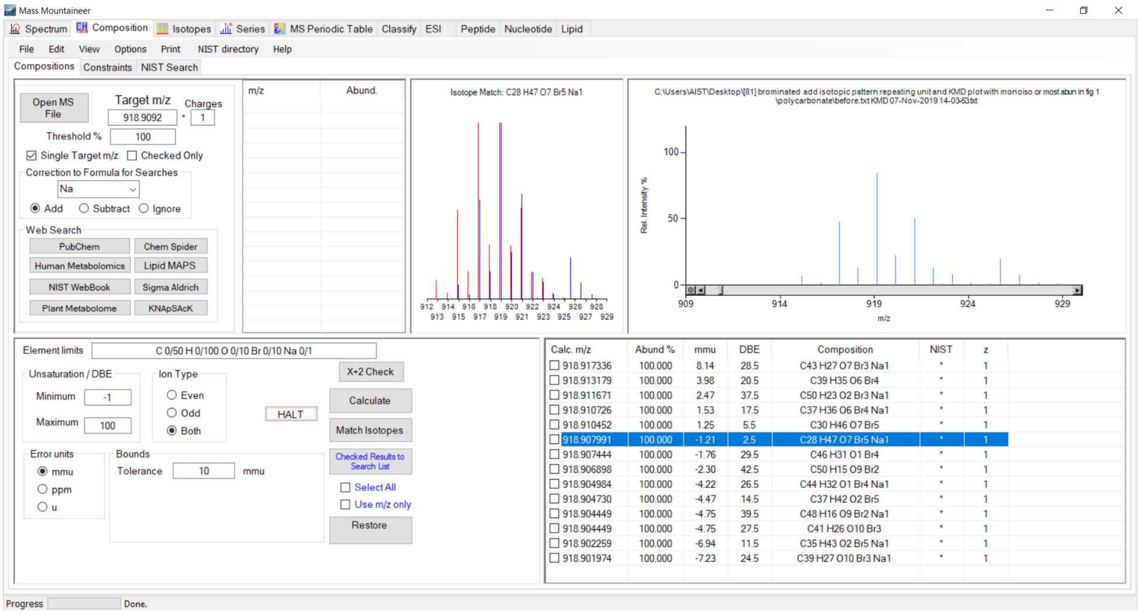

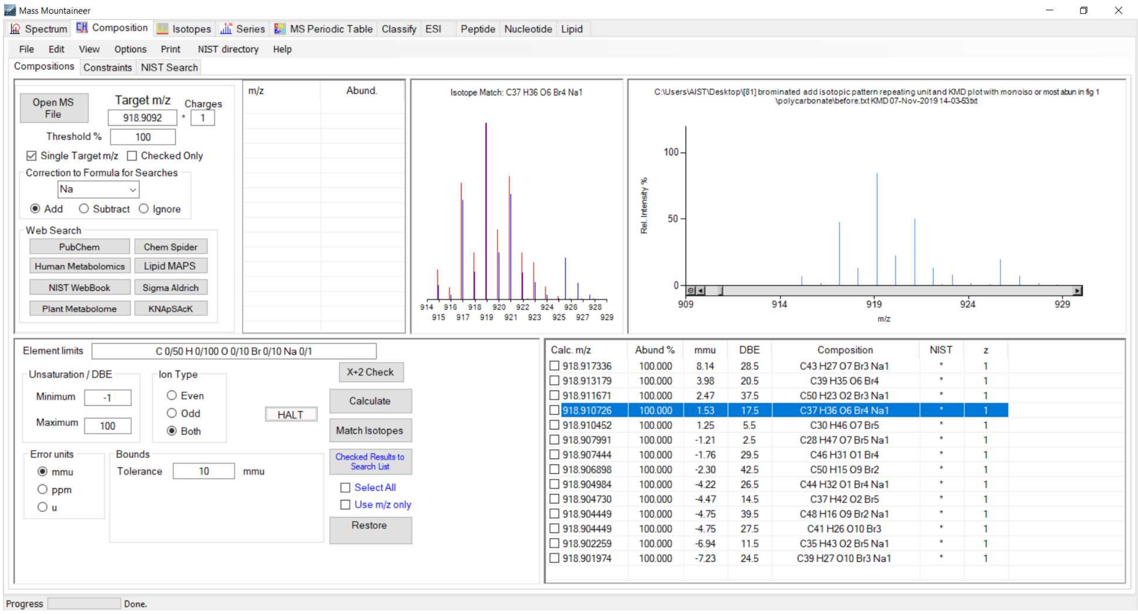

Supporting File 1. An example of a rotating plot for evaluating the repeating unit of a polymer with complex isotopic pattern

https://www.dropbox.com/s/qj6k5rqqddrbv2s/rotating%20plot.mp4?dl=0

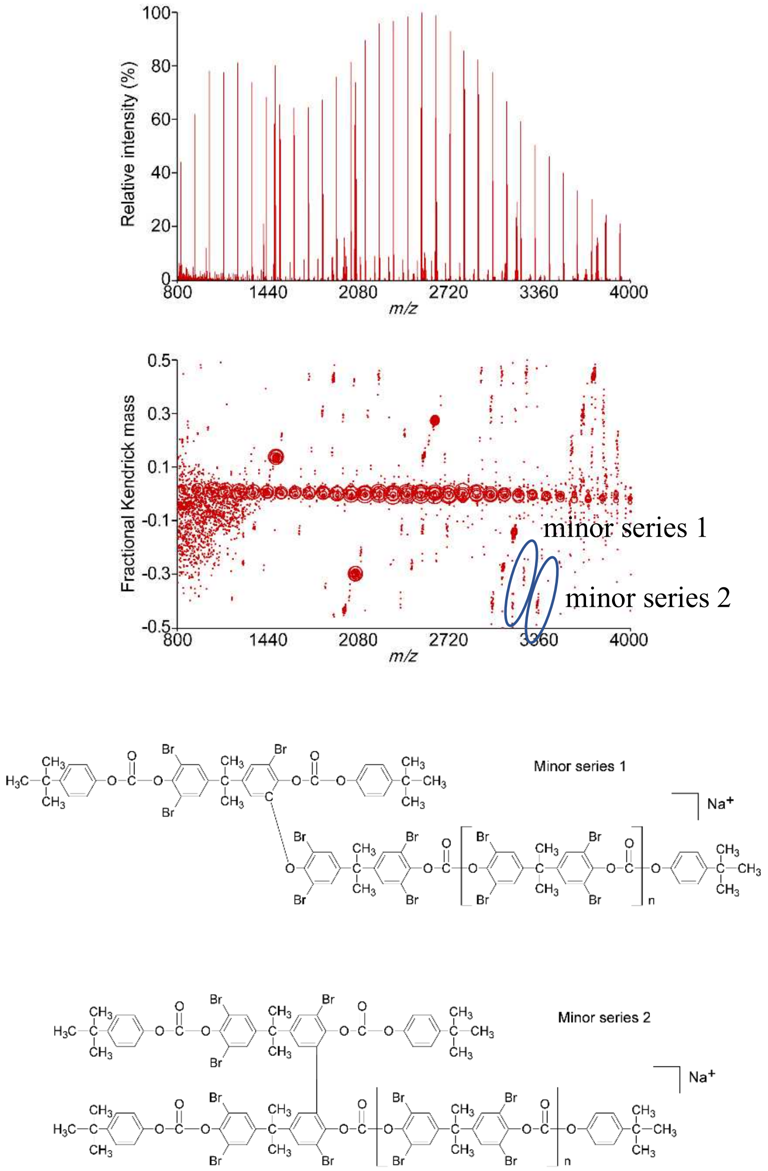

Figure S3. MALDI-spiralTOF mass spectrum of heated FRPC (T=330 degC, 5 min) with PMMA as internal calibrant. Kendrick plot using R=100.0524 and x=100. Hypothetical structures of the two minor series detected in the mass spectrum of heated FRPC.

Tables S2. Accurate mass measurements and assignments for the PMMA and FRPC main series. Errors are expressed in ppm. Peaks are selected from the Kendrick plots using kendo (polygon selection / export). Errors are indifferently computed using Excel from the peak list or kendo from the Kendrick plot (simulation + polygon selection / compute / error / plot / export).

Sodiated main series and loss of bromine from the main series

m/z_{measured}	$m/z_{theoretical}$	error	assignment: -Br +H	$m/z_{theoretical}$	error	assignment: -HBr
1171.0083	1171.0069	1.2	$[C_{37}H_{40}O_6(C_{16}H_{10}O_3Br_4)_1 + Na]^+$ = - 4Br + 4H	1170.9455	53.6	$[C_{37}H_{32}O_6(C_{16}H_{10}O_3Br_4)_1 + Na]^+$ = - 4HBr
1173.0073	1173.0055	1.5		1172.9508	48.1	
1174.0014	1174.0083	-5.8		1173.9535	40.8	
1248.9142	1248.9174	-2.5	$[C_{37}H_{39}O_6Br(C_{16}H_{10}O_3Br_4)_1 + Na]^+$ = - 3Br + 3H	1248.8665	38.2	$[C_{37}H_{33}O_6Br(C_{16}H_{10}O_3Br_4)_1 + Na]^+$ = - 3HBr
1249.9075	1249.9206	-10.5		1249.8685	31.2	
1250.9134	1250.9158	-1.9		1250.8666	37.4	
1251.9156	1251.9187	-2.5		1251.8677	38.2	
1252.9141	1252.9144	-0.2		1252.8699	35.3	
1253.9212	1253.9169	3.4		1253.8724	38.9	
1254.9083	1254.9135	-4.1		1254.8751	26.5	
1255.9080	1255.9154	-5.9		1255.8777	24.1	
1324.8229	1324.8296	-5.0	$[C_{37}H_{38}O_6Br_2(C_{16}H_{10}O_3Br_4)_1 + Na]^+$ = - 2Br + 2H	1324.7948	21.2	$[C_{37}H_{34}O_6Br_2(C_{16}H_{10}O_3Br_4)_1 + Na]^+$ = - 2HBr
1326.8232	1326.8278	-3.5		1326.7933	22.5	
1328.8249	1328.8261	-0.9		1328.7920	24.8	
1329.8243	1329.8292	-3.7		1329.7943	22.5	
1330.8240	1330.8246	-0.5		1330.7911	24.7	
1331.8241	1331.8273	-2.4		1331.7928	23.5	
1332.8203	1332.8233	-2.2		1332.7913	21.8	
1333.8254	1333.8256	-0.2		1333.7922	24.9	
1334.8224	1334.8224	0.0		1334.7943	21.0	
1335.8240	1335.8241	-0.1		1335.7967	20.4	
1336.8260	1336.8226	2.5		1336.7993	19.9	
1338.8057	1338.8256	-14.8		1338.8046	0.8	
1402.7466	1402.7401	4.6	$[C_{37}H_{37}O_6Br_3(C_{16}H_{10}O_3Br_4)_1 + Na]^+$ = - Br + H	1402.7227	17.1	$[C_{37}H_{35}O_6Br_3(C_{16}H_{10}O_3Br_4)_1 + Na]^+$ = - HBr
1403.7561	1403.7435	8.9		1403.7259	21.5	
1404.7357	1404.7383	-1.8		1404.7209	10.6	
1405.7416	1405.7416	0.0		1405.7240	12.6	
1406.7349	1406.7366	-1.2		1406.7192	11.1	
1407.7415	1407.7396	1.3		1407.7221	13.8	
1408.7355	1408.7349	0.4		1408.7177	12.6	
1409.7342	1409.7377	-2.5		1409.7203	9.9	
1410.7332	1410.7334	-0.1		1410.7165	11.9	
1411.7369	1411.7359	0.7		1411.7186	13.0	
1412.7323	1412.7321	0.2		1412.7157	11.8	
1413.7324	1413.7342	-1.3		1413.7172	10.8	
1414.7285	1414.7313	-2.0		1414.7159	9.0	
1415.7380	1415.7329	3.6		1415.7167	15.0	
1416.7305	1416.7315	-0.7		1416.7187	8.3	
1417.7277	1417.7324	-3.3		1417.7210	4.7	
1482.6485	1482.6488	-0.2	$[C_{37}H_{36}O_6Br_4(C_{16}H_{10}O_3Br_4)_1 + Na]^+$			
1483.6508	1483.6521	-0.8				
1484.6447	1484.6470	-1.6				
1485.6521	1485.6501	1.4				
1486.6466	1486.6453	0.9				
1487.6503	1487.6482	1.4				
1488.6410	1488.6436	-1.8				
1489.6454	1489.6463	-0.6				
1490.6412	1490.6422	-0.6				
1491.6463	1491.6445	1.2				
1492.6428	1492.6409	1.2				
1493.6440	1493.6429	0.8				
1494.6412	1494.6402	0.7				
1495.6431	1495.6416	1.1				
1496.6410	1496.6404	0.4				

$m/z_{measured}$	$m/z_{theoretical}$	error	assignment: -Br +H	$m/z_{theoretical}$	error	assignment : -HBr
1742.7401	1742.7386	0.9	$[C_{37}H_{40}O_6(C_{16}H_{10}O_3Br_4)_2 + Na]^+$ = - 4Br + 4H	1742.6738	38.1	$[C_{37}H_{32}O_6(C_{16}H_{10}O_3Br_4)_2 + Na]^+$ = - 4HBr
1744.7551	1744.7374	10.2		1744.6758	45.5	
1816.6515	1816.6520	-0.2	$[C_{37}H_{39}O_6Br(C_{16}H_{10}O_3Br_4)_2 + Na]^+$ = - 3Br + 3H	1816.6005	28.1	$[C_{37}H_{33}O_6Br(C_{16}H_{10}O_3Br_4)_2 + Na]^+$ = - 3HBr
1818.6450	1818.6504	-3.0		1818.5993	25.1	
1819.6421	1819.6531	-6.1		1819.6010	22.6	
1820.6641	1820.6488	8.4		1820.5984	36.1	
1821.6617	1821.6513	5.7		1821.5996	34.1	
1822.6548	1822.6474	4.0		1822.5980	31.2	
1823.6431	1823.6495	-3.5		1823.5986	24.4	
1824.6466	1824.6462	0.2		1824.5984	26.4	
1825.6306	1825.6479	-9.5		1825.5988	17.4	
1826.6296	1826.6453	-8.6		1826.6003	16.1	
1894.5683	1894.5624	3.1	$[C_{37}H_{38}O_6Br_2(C_{16}H_{10}O_3Br_4)_2 + Na]^+$ = - 2Br + 2H	1894.5279	21.4	$[C_{37}H_{34}O_6Br_2(C_{16}H_{10}O_3Br_4)_2 + Na]^+$ = - 2HBr
1895.5760	1895.5655	5.6		1895.5304	24.1	
1896.5639	1896.5608	1.7		1896.5263	19.8	
1897.5571	1897.5636	-3.4		1897.5286	15.0	
1898.5555	1898.5592	-1.9		1898.5250	16.1	
1899.5543	1899.5617	-3.9		1899.5269	14.4	
1900.5583	1900.5576	0.3		1900.5238	18.1	
1901.5675	1901.5599	4.0		1901.5253	22.2	
1902.5520	1902.5563	-2.3		1902.5229	15.3	
1903.5768	1903.5582	9.8		1903.5239	27.8	
1904.5618	1904.5551	3.5		1904.5225	20.6	
1905.5721	1905.5566	8.2		1905.5231	25.8	
1906.5476	1906.5542	-3.5		1906.5229	12.9	
1907.5685	1907.5553	6.9		1907.5233	23.7	
1908.5193	1908.5538	-18.1		1908.5247	-2.9	
1970.4745	1970.4747	-0.1	$[C_{37}H_{37}O_6Br_3(C_{16}H_{10}O_3Br_4)_2 + Na]^+$ = - Br + H	1970.4572	8.7	$[C_{37}H_{35}O_6Br_3(C_{16}H_{10}O_3Br_4)_2 + Na]^+$ = - HBr
1972.4738	1972.4729	0.5		1972.4555	9.3	
1973.4867	1973.4760	5.4		1973.4584	14.3	
1974.4691	1974.4712	-1.1		1974.4539	7.7	
1975.4773	1975.4740	1.7		1975.4565	10.5	
1976.4653	1976.4695	-2.1		1976.4523	6.6	
1977.4792	1977.4722	3.6		1977.4547	12.4	
1978.4677	1978.4679	-0.1		1978.4508	8.6	
1979.4719	1979.4703	0.8		1979.4529	9.6	
1980.4711	1980.4664	2.4		1980.4494	11.0	
1981.4655	1981.4685	-1.5		1981.4512	7.2	
1982.4653	1982.4651	0.1		1982.4483	8.6	
1983.4704	1983.4668	1.8		1983.4496	10.5	
1984.4656	1984.4639	0.8		1984.4474	9.2	
1985.4610	1985.4653	-2.2		1985.4483	6.4	
1986.4669	1986.4631	1.9		1986.4470	10.0	
1987.4628	1987.4640	-0.6		1987.4475	7.7	
1988.4538	1988.4627	-4.4		1988.4474	3.2	
1989.4605	1989.4632	-1.3		1989.4478	6.4	
2050.3842	2050.3834	0.4	$[C_{37}H_{36}O_6Br_4(C_{16}H_{10}O_3Br_4)_2 + Na]^+$			
2051.3855	2051.3865	-0.5				
2052.3871	2052.3816	2.7				
2053.3837	2053.3845	-0.4				
2054.3754	2054.3799	-2.2				
2055.3777	2055.3826	-2.4				
2056.3803	2056.3783	1.0				
2057.3831	2057.3807	1.2				
2058.3757	2058.3767	-0.5				
2059.3738	2059.3789	-2.5				
2060.3721	2060.3752	-1.5				
2061.3759	2061.3771	-0.6				
2062.3747	2062.3739	0.4				
2063.3738	2063.3755	-0.8				
2064.3731	2064.3727	0.2				
2065.3726	2065.3739	-0.6				
2066.3829	2066.3719	5.3				
2067.3672	2067.3727	-2.7				
2068.3674	2068.3715	-2.0				

$m/z_{measured}$	$m/z_{theoretical}$	error	assignment : -Br +H	$m/z_{theoretical}$	error	assignment : -HBr
2310.4741	2310.4732	0.4	$[C_{37}H_{40}O_6(C_{16}H_{10}O_3Br_4)_3 + Na]^+$ = - 4Br + 4H	2310.4057	29.6	$[C_{37}H_{32}O_6(C_{16}H_{10}O_3Br_4)_3 + Na]^+$ = - 4HBr
2312.4729	2312.4718	0.5		2312.4050	29.4	
2386.4001	2386.3850	6.3	$[C_{37}H_{39}O_6Br(C_{16}H_{10}O_3Br_4)_3 + Na]^+$ = - 3Br + 3H	2386.3336	27.9	$[C_{37}H_{33}O_6Br(C_{16}H_{10}O_3Br_4)_3 + Na]^+$ = - 3HBr
2387.3847	2387.3876	-1.2		2387.3353	20.7	
2388.3808	2388.3835	-1.1		2388.3323	20.3	
2389.3715	2389.3857	-6.0		2389.3336	15.9	
2390.3736	2390.3820	-3.5		2390.3312	17.8	
2391.3816	2391.3839	-1.0		2391.3321	20.7	
2392.3898	2392.3806	3.8		2392.3302	24.9	
2393.3700	2393.3822	-5.1		2393.3308	16.4	
2464.2681	2464.2954	-11.1	$[C_{37}H_{38}O_6Br_2(C_{16}H_{10}O_3Br_4)_3 + Na]^+$ = - 2Br + 2H	2464.2610	2.9	$[C_{37}H_{34}O_6Br_2(C_{16}H_{10}O_3Br_4)_3 + Na]^+$ = - 2HBr
2465.2858	2465.2980	-5.0		2465.2630	9.2	
2466.2923	2466.2938	-0.6		2466.2595	13.3	
2467.2818	2467.2962	-5.8		2467.2613	8.3	
2468.2830	2468.2923	-3.8		2468.2581	10.1	
2469.2901	2469.2943	-1.7		2469.2596	12.4	
2470.2917	2470.2908	0.4		2470.2568	14.1	
2471.2820	2471.2926	-4.3		2471.2579	9.7	
2472.2897	2472.2894	0.1		2472.2556	13.8	
2473.2862	2473.2909	-1.9		2473.2565	12.0	
2474.2714	2474.2881	-6.8		2474.2547	6.7	
2475.2854	2475.2892	-1.6		2475.2552	12.2	
2476.2653	2476.2869	-8.8		2476.2540	4.6	
2477.2740	2477.2878	-5.6		2477.2542	8.0	
2478.2887	2478.2860	1.1		2478.2537	14.1	
2479.2978	2479.2865	4.6		2479.2539	17.7	
2540.1986	2540.2075	-3.5	$[C_{37}H_{37}O_6Br_3(C_{16}H_{10}O_3Br_4)_3 + Na]^+$ = - Br + H	2540.1902	3.3	$[C_{37}H_{35}O_6Br_3(C_{16}H_{10}O_3Br_4)_3 + Na]^+$ = - HBr
2541.1854	2541.2104	-9.8		2541.1928	-2.9	
2542.1956	2542.2059	-4.0		2542.1886	2.8	
2543.2003	2543.2085	-3.2		2543.1910	3.7	
2544.2109	2544.2042	2.6		2544.1869	9.4	
2545.1927	2545.2066	-5.5		2545.1891	1.4	
2546.1979	2546.2026	-1.8		2546.1854	4.9	
2547.1918	2547.2048	-5.1		2547.1873	1.7	
2548.1916	2548.2010	-3.7		2548.1839	3.0	
2549.1916	2549.2030	-4.5		2549.1855	2.4	
2550.1918	2550.1996	-3.1		2550.1825	3.6	
2551.1922	2551.2012	-3.5		2551.1839	3.3	
2552.1928	2552.1982	-2.1		2552.1812	4.5	
2553.1936	2553.1995	-2.3		2553.1823	4.4	
2554.1887	2554.1969	-3.2		2554.1801	3.4	
2555.1841	2555.1979	-5.4		2555.1808	1.3	
2556.1913	2556.1957	-1.7		2556.1791	4.8	
2557.1870	2557.1964	-3.7		2557.1795	2.9	
2558.1830	2558.1948	-4.6		2558.1785	1.8	
2559.2024	2559.1952	2.8		2559.1786	9.3	
2560.1696	2560.1941	-9.6		2560.1782	-3.3	
2618.1119	2618.1180	-2.3	$[C_{37}H_{36}O_6Br_4(C_{16}H_{10}O_3Br_4)_3 + Na]^+$			
2619.1314	2619.1209	4.0				
2620.1099	2620.1163	-2.4				
2621.1180	2621.1190	-0.4				
2622.1028	2622.1146	-4.5				
2623.1054	2623.1171	-4.5				
2624.1023	2624.1129	-4.1				
2625.0993	2625.1152	-6.0				
2626.1025	2626.1114	-3.4				
2627.1000	2627.1134	-5.1				
2628.1036	2628.1098	-2.4				
2629.1014	2629.1116	-3.9				
2630.0995	2630.1083	-3.4				
2631.0977	2631.1098	-4.6				
2632.0961	2632.1069	-4.1				
2633.0947	2633.1081	-5.1				
2634.0935	2634.1057	-4.6				
2635.0984	2635.1066	-3.1				
2636.0976	2636.1045	-2.6				
2637.0969	2637.1051	-3.1				
2638.0965	2638.1036	-2.7				
2639.0903	2639.1039	-5.1				

m/z_{measured}	$m/z_{theoretical}$	error	assignment : -Br +H	$m/z_{theoretical}$	error	assignment : -HBr
2958.1279	2958.1167	3.8	$[C_{37}H_{39}O_6Br(C_{16}H_{10}O_3Br_4)_4 + Na]^+$ = - 3Br + 3H	2958.0654	21.1	$[C_{37}H_{33}O_6Br(C_{16}H_{10}O_3Br_4)_4 + Na]^+$ = - 3HBr
2959.0989	2959.1184	-6.6		2959.0664	11.0	
2960.1013	2960.1152	-4.7		2960.0642	12.5	
2962.0941	2962.1138	-6.6		2962.0630	10.5	
2964.0813	2964.1124	-10.5		2964.0619	6.5	
2965.0909	2965.1133	-7.6		2965.0621	9.7	
3033.9969	3034.0286	-10.5	$[C_{37}H_{38}O_6Br_2(C_{16}H_{10}O_3Br_4)_4 + Na]^+$ = - 2Br + 2H	3033.9942	0.9	$[C_{37}H_{34}O_6Br_2(C_{16}H_{10}O_3Br_4)_4 + Na]^+$ = - 2HBr
3034.9929	3035.0306	-12.5		3034.9957	-0.9	
3035.9763	3036.0270	-16.7		3035.9927	-5.4	
3037.0107	3037.0288	-6.0		3036.9940	5.5	
3038.0326	3038.0255	2.4		3037.9912	13.6	
3039.0102	3039.0270	-5.5		3038.9923	5.9	
3040.0007	3040.0240	-7.7		3039.9899	3.6	
3041.0167	3041.0253	-2.8		3040.9907	8.6	
3042.0202	3042.0225	-0.8		3041.9886	10.4	
3043.0239	3043.0236	0.1		3042.9891	11.4	
3044.0087	3044.0212	-4.1		3043.9874	7.0	
3045.0126	3045.0220	-3.1		3044.9877	8.2	
3045.9850	3046.0199	-11.5		3045.9864	-0.5	
3046.9893	3047.0205	-10.2		3046.9865	0.9	
3047.9620	3048.0187	-18.6		3047.9855	-7.7	
3049.0048	3049.0190	-4.7		3048.9855	6.3	
3108.9126	3108.9448	-10.4	$[C_{37}H_{37}O_6Br_3(C_{16}H_{10}O_3Br_4)_4 + Na]^+$ = - Br + H	3108.9273	-4.7	$[C_{37}H_{35}O_6Br_3(C_{16}H_{10}O_3Br_4)_4 + Na]^+$ = - HBr
3109.9465	3109.9406	1.9		3109.9233	7.5	
3110.9035	3110.9430	-12.7		3110.9254	-7.1	
3111.9184	3111.9389	-6.6		3111.9217	-1.0	
3112.9014	3112.9411	-12.7		3112.9236	-7.1	
3113.9424	3113.9373	1.6		3113.9201	7.2	
3114.9065	3114.9393	-10.5		3114.9218	-4.9	
3115.8964	3115.9358	-12.6		3115.9186	-7.1	
3116.9186	3116.9374	-6.1		3116.9200	-0.5	
3117.9152	3117.9342	-6.1		3117.9171	-0.6	
3118.9120	3118.9357	-7.6		3118.9183	-2.0	
3119.9090	3119.9327	-7.6		3119.9157	-2.1	
3120.9061	3120.9339	-8.9		3120.9166	-3.3	
3121.9099	3121.9313	-6.9		3121.9143	-1.4	
3122.9009	3122.9323	-10.0		3122.9150	-4.5	
3123.9049	3123.9300	-8.0		3123.9130	-2.6	
3124.8963	3124.9306	-11.0		3124.9135	-5.5	
3125.9071	3125.9287	-6.9		3125.9119	-1.5	
3185.8209	3185.8527	-10.0	$[C_{37}H_{36}O_6Br_4(C_{16}H_{10}O_3Br_4)_4 + Na]^+$			
3186.8480	3186.8553	-2.3				
3187.8492	3187.8510	-0.6				
3188.8376	3188.8534	-4.9				
3189.8262	3189.8493	-7.2				
3190.8345	3190.8516	-5.4				
3191.8299	3191.8477	-5.6				
3192.8319	3192.8497	-5.6				
3193.8146	3193.8461	-9.9				
3194.8365	3194.8479	-3.6				
3195.8129	3195.8445	-9.9				
3196.8221	3196.8461	-7.5				
3197.8185	3197.8430	-7.7				
3198.8214	3198.8443	-7.1				
3199.8116	3199.8415	-9.4				
3200.8149	3200.8426	-8.7				
3201.8183	3201.8401	-6.8				
3202.8219	3202.8409	-5.9				
3203.8127	3203.8387	-8.1				
3204.8101	3204.8393	-9.1				
3205.8142	3205.8374	-7.3				
3206.7989	3206.8378	-12.1				
3207.7967	3207.8363	-12.3				
3208.8143	3208.8364	-6.9				

$m/z_{measured}$	$m/z_{theoretical}$	error	assignment : -Br +H	$m/z_{theoretical}$	error	assignment : -HBr
3603.7265	3603.7617	-9.8		3603.7274	-0.2	
3604.7429	3604.7633	-5.7		3604.7285	4.0	
3605.7663	3605.7602	1.7		3605.7259	11.2	
3606.7068	3606.7616	-15.2		3606.7268	-5.5	
3607.7374	3607.7587	-5.9		3607.7244	3.6	
3608.7405	3608.7598	-5.4		3608.7251	4.3	
3609.7298	3609.7572	-7.6		3609.7230	1.9	
3610.7055	3610.7581	-14.6	$[C_{37}H_{38}O_6Br_2(C_{16}H_{10}O_3Br_4)_5 + Na]^+$ = - 2Br + 2H	3610.7235	-5.0	$[C_{37}H_{34}O_6Br_2(C_{16}H_{10}O_3Br_4)_5 + Na]^+$ = - 2HBr
3611.7228	3611.7557	-9.1		3611.7216	0.3	
3612.7403	3612.7564	-4.5		3612.7219	5.1	
3613.7025	3613.7543	-14.3		3613.7203	-4.9	
3614.7341	3614.7548	-5.7		3614.7205	3.8	
3615.7381	3615.7529	-4.1		3615.7191	5.3	
3616.7215	3616.7532	-8.8		3616.7191	0.7	
3617.7258	3617.7516	-7.1		3617.7180	2.2	
3619.7210	3619.7504	-8.1		3619.7171	1.1	
3679.6717	3679.6737	-0.5		3679.6565	4.2	
3680.6358	3680.6756	-10.8		3680.6581	-6.1	
3682.6134	3682.6738	-16.4		3682.6563	-11.7	
3683.6547	3683.6705	-4.3		3683.6533	0.4	
3684.6403	3684.6720	-8.6		3684.6545	-3.9	
3685.6331	3685.6690	-9.7		3685.6518	-5.1	
3686.6399	3686.6702	-8.2		3686.6528	-3.5	
3687.6189	3687.6674	-13.2		3687.6503	-8.5	
3688.6400	3688.6684	-7.7		3688.6511	-3.0	
3689.6193	3689.6659	-12.6		3689.6488	-8.0	
3690.6477	3690.6667	-5.2	$[C_{37}H_{37}O_6Br_3(C_{16}H_{10}O_3Br_4)_5 + Na]^+$ = - Br + H	3690.6494	-0.5	$[C_{37}H_{35}O_6Br_3(C_{16}H_{10}O_3Br_4)_5 + Na]^+$ = - HBr
3691.6202	3691.6645	-12.0		3691.6474	-7.4	
3692.6279	3692.6651	-10.1		3692.6478	-5.4	
3693.6357	3693.6630	-7.4		3693.6460	-2.8	
3694.6506	3694.6634	-3.5		3694.6463	1.2	
3695.6727	3695.6617	3.0		3695.6447	7.6	
3696.6039	3696.6619	-15.7		3696.6448	-11.1	
3697.6192	3697.6604	-11.1		3697.6435	-6.6	
3698.6137	3698.6605	-12.6		3698.6435	-8.1	
3699.6223	3699.6592	-10.0		3699.6424	-5.4	
3700.5750	3700.6591	-22.7		3700.6423	-18.2	
3701.6469	3701.6581	-3.0		3701.6415	1.5	
3755.5418	3755.5858	-11.7				
3756.5228	3756.5879	-17.3				
3757.5464	3757.5841	-10.0				
3758.5277	3758.5861	-15.5				
3759.5232	3759.5825	-15.8				
3760.5331	3760.5842	-13.6				
3761.5359	3761.5809	-11.9				
3762.5248	3762.5824	-15.3				
3763.5351	3763.5793	-11.7				
3764.5313	3764.5806	-13.1				
3765.5347	3765.5777	-11.4				
3766.5383	3766.5788	-10.8	$[C_{37}H_{36}O_6Br_4(C_{16}H_{10}O_3Br_4)_5 + Na]^+$			
3767.5208	3767.5762	-14.7				
3768.5317	3768.5771	-12.0				
3769.5215	3769.5747	-14.1				
3770.5256	3770.5754	-13.2				
3771.5298	3771.5732	-11.5				
3772.5271	3772.5737	-12.4				
3773.5033	3773.5718	-18.2				
3774.5150	3774.5721	-15.1				
3775.5268	3775.5704	-11.6				
3776.5317	3776.5706	-10.3				
3777.5013	3777.5691	-18.0				
3778.5348	3778.5691	-9.1				

Minor series 1

Main series			
$m/z_{measured}$	$m/z_{theoretical}$	error	assignment
1888.8331	1888.8291	2.1	$[C_{63}H_{62}Br_4O_{10}(C_{16}H_{10}O_3Br_4)_1 + Na]^+$ = -3Br + 3H
1890.8357	1890.8279	4.1	
1891.8474	1891.8299	9.2	
1892.8193	1892.8268	-3.9	
1893.8365	1893.8284	4.3	
1966.7597	1966.7395	10.3	$[C_{63}H_{61}Br_5O_{10}(C_{16}H_{10}O_3Br_4)_1 + Na]^+$ = -2Br + 2H
1968.7367	1968.7381	-0.7	
1970.7352	1970.7368	-0.8	
1971.7476	1971.7386	4.6	
1972.7398	1972.7357	2.1	
2046.6677	2046.6483	9.5	$[C_{63}H_{60}Br_6O_{10}(C_{16}H_{10}O_3Br_4)_1 + Na]^+$ = -Br + H
2048.6480	2048.6469	0.5	
2049.6542	2049.6489	2.6	
2050.6449	2050.6457	-0.4	
2051.6515	2051.6473	2.1	
not detected			$[C_{63}H_{59}Br_7O_{10}(C_{16}H_{10}O_3Br_4)_1 + Na]^+$
2380.6596	2380.6522	3.1	$[C_{63}H_{63}Br_3O_{10}(C_{16}H_{10}O_3Br_4)_2 + Na]^+$ = -4Br + 4H
2381.6711	2381.6539	7.2	
2382.6154	2382.6509	-14.9	
2384.6396	2384.6499	-4.3	
2458.5659	2458.5624	1.4	$[C_{63}H_{62}Br_4O_{10}(C_{16}H_{10}O_3Br_4)_2 + Na]^+$ = -3Br + 3H
2459.5482	2459.5643	-6.6	
2460.5707	2460.5611	3.9	
2461.5705	2461.5626	3.2	
2462.5362	2462.5598	-9.6	
2463.5707	2463.5610	3.9	
2464.5597	2464.5588	0.4	
2465.5660	2465.5596	2.6	
2466.5439	2466.5579	-5.6	
2467.5507	2467.5584	-3.1	
2534.4671	2534.4742	-2.8	$[C_{63}H_{61}Br_5O_{10}(C_{16}H_{10}O_3Br_4)_2 + Na]^+$ = -2Br + 2H
2535.4761	2535.4765	-0.2	
2536.4562	2536.4727	-6.5	
2537.4597	2537.4747	-5.9	
2538.4692	2538.4713	-0.8	
2539.4499	2539.4730	-9.1	
2540.4714	2540.4699	0.6	
2541.4757	2541.4713	1.7	
2542.4802	2542.4687	4.5	
2543.4558	2543.4697	-5.5	
2544.4491	2544.4676	-7.3	
2546.4653	2546.4668	-0.6	
2547.4301	2547.4671	-14.6	
not detected			$[C_{63}H_{60}Br_6O_{10}(C_{16}H_{10}O_3Br_4)_2 + Na]^+$ = -Br + H
not detected			$[C_{63}H_{59}Br_7O_{10}(C_{16}H_{10}O_3Br_4)_2 + Na]^+$

Main series			
$m/z_{measured}$	$m/z_{theoretical}$	error	assignment
2950.3474	2950.3854	-12.9	$[C_{63}H_{63}Br_3O_{10}(C_{16}H_{10}O_3Br_4)_3 + Na]^+$ = -4Br + 4H
2951.3358	2951.3867	-17.2	
3025.2683	3025.3005	-10.7	$[C_{63}H_{62}Br_4O_{10}(C_{16}H_{10}O_3Br_4)_3 + Na]^+$ = -3Br + 3H
3027.3209	3027.2988	7.3	
3028.2778	3028.2956	-5.9	
3029.2792	3029.2971	-5.9	
3030.2681	3030.2943	-8.6	
3031.2761	3031.2954	-6.4	
3032.3034	3032.2930	3.4	
3033.3181	3033.2938	8.0	
3034.2760	3034.2918	-5.2	
3035.2466	3035.2923	-15.0	
3038.2357	3038.2897	-17.7	
3039.2261	3039.2897	-21.0	
3104.1498	3104.2074	-18.5	$[C_{63}H_{61}Br_5O_{10}(C_{16}H_{10}O_3Br_4)_3 + Na]^+$ = -2Br + 2H
3105.1573	3105.2092	-16.7	
3106.1906	3106.2059	-4.9	
3107.1727	3107.2074	-11.2	
3108.2063	3108.2045	0.6	
3109.1694	3109.2057	-11.7	
3110.1777	3110.2031	-8.2	
3111.1861	3111.2041	-5.8	
3112.1883	3112.2018	-4.4	
3113.1713	3113.2025	-10.0	
3114.1866	3114.2006	-4.5	
3115.1314	3115.2010	-22.3	
3183.0914	3183.1196	-8.9	$[C_{63}H_{60}Br_6O_{10}(C_{16}H_{10}O_3Br_4)_3 + Na]^+$ = -Br + H
3184.0855	3184.1162	-9.6	
3185.0734	3185.1178	-14.0	
3186.1264	3186.1147	3.6	
3187.0755	3187.1161	-12.7	
3188.0833	3188.1133	-9.4	
3189.0978	3189.1144	-5.2	
3190.0799	3190.1119	-10.0	
3191.0426	3191.1128	-22.0	
3193.0466	3193.1112	-20.2	
3264.0043	3264.0250	-6.3	$[C_{63}H_{59}Br_7O_{10}(C_{16}H_{10}O_3Br_4)_3 + Na]^+$
3265.0308	3265.0265	1.3	
3266.0509	3266.0235	8.4	
3268.0256	3268.0221	1.1	
3270.0010	3270.0207	-6.0	
3271.0218	3271.0214	0.1	
3272.0362	3272.0194	5.1	

Minor series 2

Main series			
$m/z_{measured}$	$m/z_{theoretical}$	error	assignment
2142.8287	2142.824	2.4	$[C_{74}H_{73}Br_5O_{12}(C_{16}H_{10}O_3Br_4)_1 + Na]^+$ = -Br + H
2146.8128	2146.821	-3.9	
2147.8374	2147.823	6.9	
2148.8142	2148.82	-2.8	
2149.8394	2149.821	8.4	
2220.7171	2220.734	-7.5	$[C_{74}H_{72}Br_6O_{12}(C_{16}H_{10}O_3Br_4)_1 + Na]^+$
2222.7202	2222.732	-5.5	
2223.7247	2223.735	-4.4	
2224.7295	2224.731	-0.7	
2225.7400	2225.733	3.2	
2226.7181	2226.73	-5.4	
2227.7562	2227.731	11.1	
2228.7184	2228.729	-4.8	
2229.7080	2229.73	-9.9	
2231.7368	2231.729	3.5	
2637.6292	2637.6467	-6.6	$[C_{74}H_{74}Br_4O_{12}(C_{16}H_{10}O_3Br_4)_2 + Na]^+$ = -2Br + 2H
2638.6052	2638.6442	-14.8	
2640.6347	2640.6432	-3.2	
2710.5322	2710.5582	-9.6	$[C_{74}H_{73}Br_5O_{12}(C_{16}H_{10}O_3Br_4)_2 + Na]^+$ = -Br + H
2711.5336	2711.5604	-9.9	
2712.5472	2712.5568	-3.6	
2713.5429	2713.5586	-5.8	
2714.5509	2714.5555	-1.7	
2716.5553	2716.5543	0.4	
2717.5458	2717.5554	-3.5	
2718.5485	2718.5531	-1.7	
2719.5633	2719.5539	3.5	
2720.5183	2720.5521	-12.4	
2721.5456	2721.5526	-2.6	
2788.4655	2788.4686	-1.1	$[C_{74}H_{72}Br_6O_{12}(C_{16}H_{10}O_3Br_4)_2 + Na]^+$
2789.4751	2789.4708	1.5	
2790.4545	2790.4671	-4.5	
2791.4644	2791.4690	-1.6	
2792.4502	2792.4657	-5.6	
2793.4545	2793.4673	-4.6	
2794.4528	2794.4644	-4.2	
2795.4452	2795.4657	-7.3	
2796.4560	2796.4632	-2.5	
2797.4610	2797.4641	-1.1	
2798.4539	2798.4620	-2.9	
2799.4592	2799.4627	-1.2	
2801.4643	2801.4614	1.0	
2802.4701	2802.4602	3.5	
2803.4457	2803.4603	-5.2	

Main series			
$m/z_{measured}$	$m/z_{theoretical}$	error	assignment
3204.3603	3204.3799	-6.1	$[C_{74}H_{74}Br_4O_{12}(C_{16}H_{10}O_3Br_4)_3 + Na]^+$ = -2Br + 2H
3208.4099	3208.3773	10.1	
3209.3689	3209.3779	-2.8	
3278.2644	3278.2930	-8.7	$[C_{74}H_{73}Br_5O_{12}(C_{16}H_{10}O_3Br_4)_3 + Na]^+$ = -Br + H
3280.2626	3280.2916	-8.8	
3281.3048	3281.2931	3.6	
3282.2879	3282.2901	-0.7	
3283.2776	3283.2914	-4.2	
3284.2675	3284.2888	-6.5	
3285.2708	3285.2898	-5.8	
3286.2742	3286.2875	-4.0	
3287.2447	3287.2882	-13.2	
3288.2550	3288.2862	-9.5	
3289.2457	3289.2867	-12.5	
3290.2695	3290.2850	-4.7	
3291.2605	3291.2853	-7.5	
3292.2251	3292.2840	-17.9	
3358.2012	3358.2019	-0.2	$[C_{74}H_{72}Br_6O_{12}(C_{16}H_{10}O_3Br_4)_3 + Na]^+$
3359.1757	3359.2036	-8.3	
3360.1636	3360.2004	-11.0	
3361.1850	3361.2018	-5.0	
3362.1866	3362.1990	-3.7	
3363.1817	3363.2001	-5.5	
3364.1768	3364.1976	-6.2	
3365.1722	3365.1985	-7.8	
3366.1677	3366.1963	-8.5	
3367.1633	3367.1969	-10.0	
3368.1725	3368.1950	-6.7	
3369.1684	3369.1954	-8.0	
3370.1511	3370.1939	-12.7	
3371.1607	3371.1940	-9.9	
3372.2106	3372.1928	5.3	
3373.2004	3373.1928	2.3	

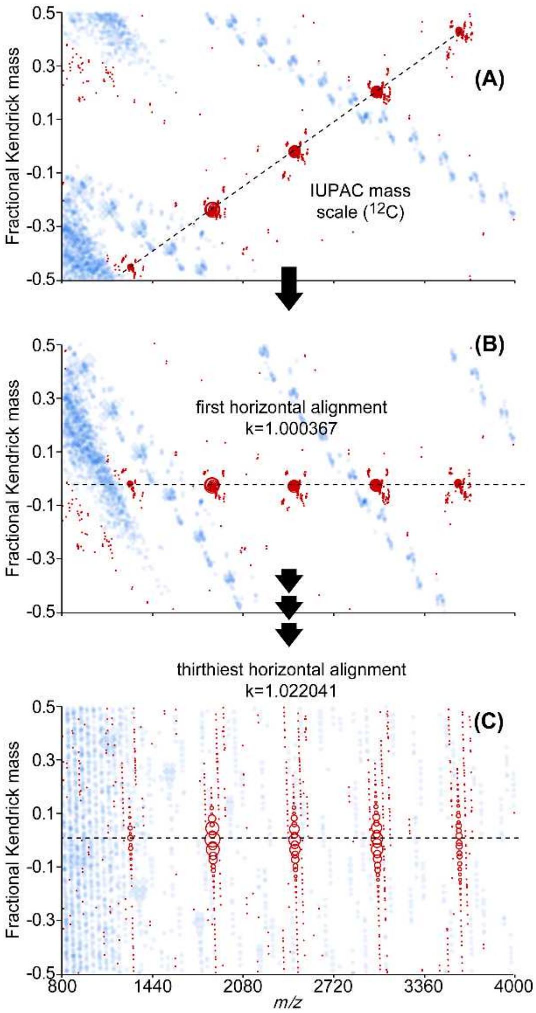

Figure S4. (A) Kendrick plot from the filtered peak list of the PBT resin using ^{12}C. (B) First horizontal alignment. (C) Thirteenth horizontal alignment with considerable expansion of the isotopic pattern: the mass of R is evaluated ab initio.

Figure S5. screenshots of Mass Mountaineer for the computation of elemental compositions from the most abundant isotope

a) repeating unit using the mass difference, the single rotation and the double rotation

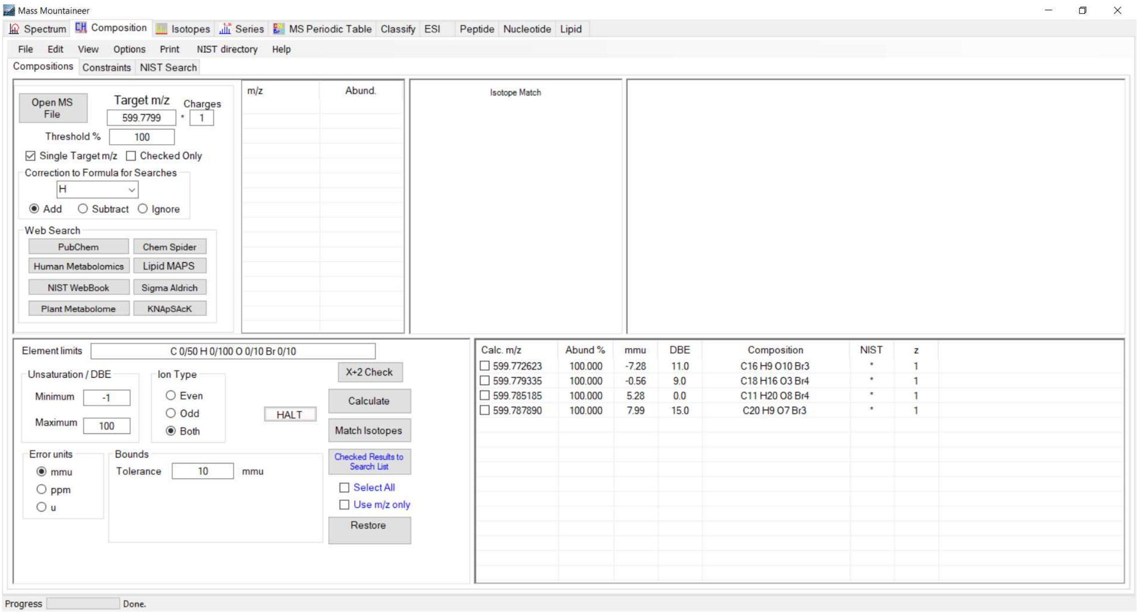

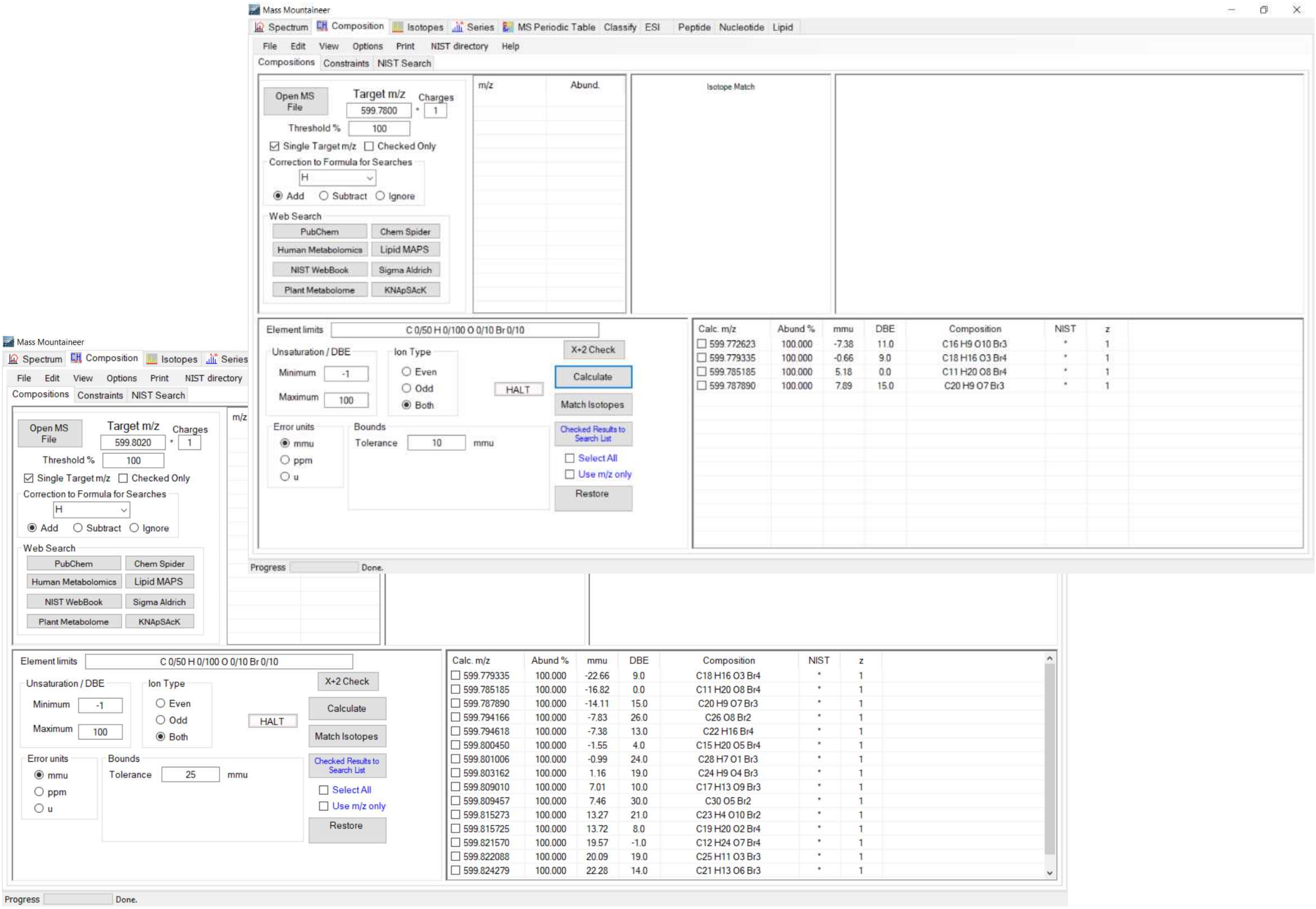

b) First oligomer

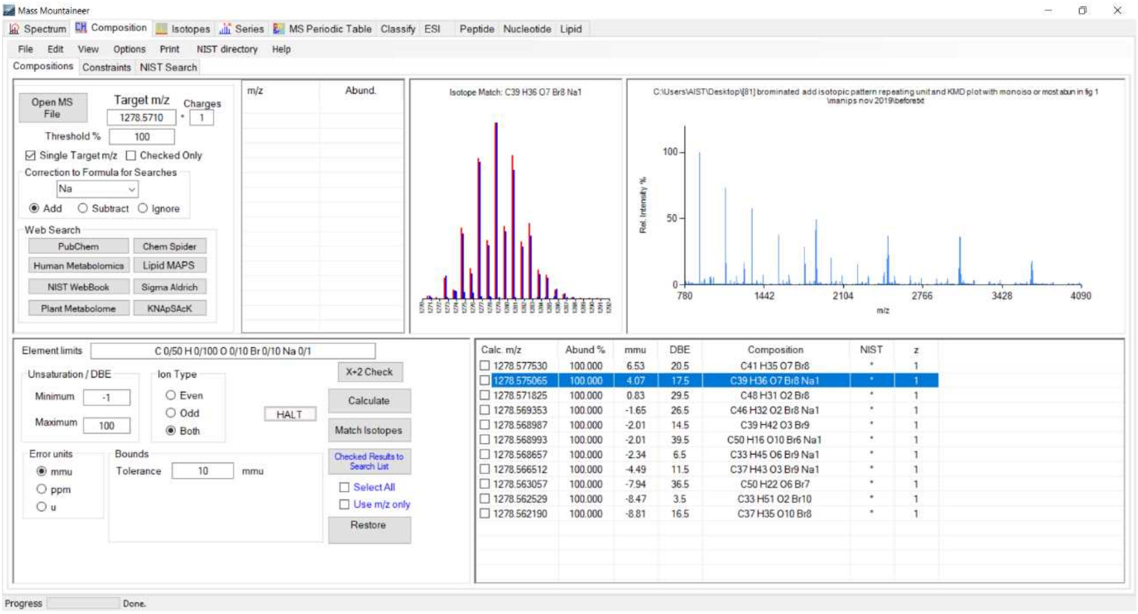

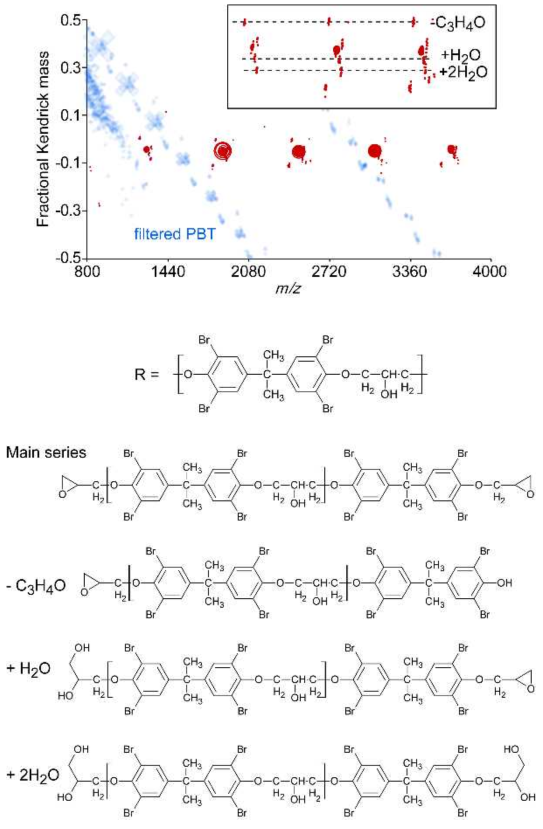

Figure S6. Kendrick plot of the filtered peak list of the PBT resin using $C_{18}H_{16}O_3Br_4$ as the base unit. Structures of the minor series.

Tables S3. Accurate mass measurements and assignments for the main series (PBT and brominated flamed retardant) detected in the PBT / flame retardant / Sb2O3 sample. Errors are expressed in ppm. Peaks are selected from the Kendrick plots using kendo (polygon selection / export). Errors are indifferently computed using Excel from the peak list or kendo from the Kendrick plot (simulation + polygon selection / compute / error / plot / export).

sodiated cyclic PBT used as internal calibrant

$m/z_{measured}$	$m/z_{theoretical}$	error	assignment
903.2783	-	-	$[(C_{12}H_{12}O_4)_4 + Na]^+$
1123.3564	-	-	$[(C_{12}H_{12}O_4)_5 + Na]^+$
1343.4304	-	-	$[(C_{12}H_{12}O_4)_6 + Na]^+$
1563.5060	-	-	$[(C_{12}H_{12}O_4)_7 + Na]^+$
1783.5785	-	--	$[(C_{12}H_{12}O_4)_8 + Na]^+$
2003.6548	-	-	$[(C_{12}H_{12}O_4)_9 + Na]^+$
2223.7271	-	-	$[(C_{12}H_{12}O_4)_{10} + Na]^+$
2443.8010	-	-	$[(C_{12}H_{12}O_4)_{11} + Na]^+$
2663.8740	-	-	$[(C_{12}H_{12}O_4)_{12} + Na]^+$
2883.9443	-	-	$[(C_{12}H_{12}O_4)_{13} + Na]^+$
3104.0149	-	-	$[(C_{12}H_{12}O_4)_{14} + Na]^+$
3324.0903	-	-	$[(C_{12}H_{12}O_4)_{15} + Na]^+$
3544.1563	-	-	$[(C_{12}H_{12}O_4)_{16} + Na]^+$
3764.2341	-	-	$[(C_{12}H_{12}O_4)_{17} + Na]^+$

Sodiated $(C_4H_{10}O_2)$-PBT

$m/z_{measured}$	$m/z_{theoretical}$	error	assignment
993.3480	993.3515	-3.6	$[C_4H_{10}O_2(C_{12}H_{12}O_4)_4 + Na]^+$
1213.4232	1213.4251	-1.6	$[C_4H_{10}O_2(C_{12}H_{12}O_4)_5 + Na]^+$
1433.4962	1433.4987	-1.7	$[C_4H_{10}O_2(C_{12}H_{12}O_4)_6 + Na]^+$
1653.5732	1653.5722	0.6	$[C_4H_{10}O_2(C_{12}H_{12}O_4)_7 + Na]^+$
1873.6478	1873.6458	1.1	$[C_4H_{10}O_2(C_{12}H_{12}O_4)_8 + Na]^+$
2093.7217	2093.7193	1.1	$[C_4H_{10}O_2(C_{12}H_{12}O_4)_9 + Na]^+$
2313.7917	2313.7929	-0.5	$[C_4H_{10}O_2(C_{12}H_{12}O_4)_{10} + Na]^+$
2533.8704	2533.8664	1.6	$[C_4H_{10}O_2(C_{12}H_{12}O_4)_{11} + Na]^+$
2753.9395	2753.9400	-0.2	$[C_4H_{10}O_2(C_{12}H_{12}O_4)_{12} + Na]^+$
2974.0134	2974.0136	-0.1	$[C_4H_{10}O_2(C_{12}H_{12}O_4)_{13} + Na]^+$
3194.0847	3194.0871	-0.7	$[C_4H_{10}O_2(C_{12}H_{12}O_4)_{14} + Na]^+$
3414.1594	3414.1607	-0.4	$[C_4H_{10}O_2(C_{12}H_{12}O_4)_{15} + Na]^+$
3634.2229	3634.2342	-3.1	$[C_4H_{10}O_2(C_{12}H_{12}O_4)_{16} + Na]^+$

Sodiated ($C_{14}H_{12}O_5$)-PBT

$m/z_{measured}$	$m/z_{theoretical}$	error	assignment
943.2698	943.2784	-9.1	$[C_{14}H_{12}O_5(C_{12}H_{12}O_4)_3 + Na]^+$
1163.3472	1163.3519	-4.1	$[C_{14}H_{12}O_5(C_{12}H_{12}O_4)_4 + Na]^+$
1383.4225	1383.4255	-2.2	$[C_{14}H_{12}O_5(C_{12}H_{12}O_4)_5 + Na]^+$
1603.4982	1603.4990	-0.5	$[C_{14}H_{12}O_5(C_{12}H_{12}O_4)_6 + Na]^+$
1823.5693	1823.5726	-1.8	$[C_{14}H_{12}O_5(C_{12}H_{12}O_4)_7 + Na]^+$
2043.6477	2043.6462	0.7	$[C_{14}H_{12}O_5(C_{12}H_{12}O_4)_8 + Na]^+$
2263.7166	2263.7197	-1.4	$[C_{14}H_{12}O_5(C_{12}H_{12}O_4)_9 + Na]^+$
2483.7964	2483.7933	1.2	$[C_{14}H_{12}O_5(C_{12}H_{12}O_4)_{10} + Na]^+$
2703.8633	2703.8668	-1.3	$[C_{14}H_{12}O_5(C_{12}H_{12}O_4)_{11} + Na]^+$
2923.9397	2923.9404	-0.2	$[C_{14}H_{12}O_5(C_{12}H_{12}O_4)_{12} + Na]^+$

Sodiated (C_4H_8O)-PBT, Na

$m/z_{measured}$	$m/z_{theoretical}$	error	assignment
975.3348	975.3410	-6.4	$[C_4H_8O(C_{12}H_{12}O_4)_4 + Na]^+$
1195.4124	1195.4145	-1.8	$[C_4H_8O(C_{12}H_{12}O_4)_5 + Na]^+$
1415.4867	1415.4881	-1.0	$[C_4H_8O(C_{12}H_{12}O_4)_6 + Na]^+$
1635.5630	1635.5616	0.8	$[C_4H_8O(C_{12}H_{12}O_4)_7 + Na]^+$
1855.6342	1855.6352	-0.6	$[C_4H_8O(C_{12}H_{12}O_4)_8 + Na]^+$
2075.7051	2075.7088	-1.8	$[C_4H_8O(C_{12}H_{12}O_4)_9 + Na]^+$
2295.7825	2295.7823	0.1	$[C_4H_8O(C_{12}H_{12}O_4)_{10} + Na]^+$
2515.8542	2515.8559	-0.7	$[C_4H_8O(C_{12}H_{12}O_4)_{11} + Na]^+$
2735.9270	2735.9294	-0.9	$[C_4H_8O(C_{12}H_{12}O_4)_{12} + Na]^+$

Sodiated main series

$m/z_{measured}$	$m/z_{theoretical}$	error	assignment
1270.5864	1270.5826	3.0	$[C_{21}H_{20}O_4Br_4(C_{18}H_{16}O_3Br_4)_1 + Na]^+$
N.D.	1271.5860	N.D.	
1272.5784	1272.5806	-1.8	
1273.5769	1273.5839	-5.5	
1274.5759	1274.5787	-2.2	
1275.5795	1275.5820	-2.0	
1276.5751	1276.5769	-1.4	
1277.5752	1277.5800	-3.8	
1278.5717	1278.5751	-2.7	
1279.5768	1279.5780	-1.0	
1280.5698	1280.5734	-2.8	
1281.5715	1281.5762	-3.6	
1282.5696	1282.5719	-1.8	
1283.5721	1283.5744	-1.7	
1284.5668	1284.5707	-3.1	
1868.3671	1868.3640	1.7	$[C_{21}H_{20}O_4Br_4(C_{18}H_{16}O_3Br_4)_2 + Na]^+$
1869.3678	1869.3673	0.3	
1870.3639	1870.3621	1.0	
1871.3702	1871.3653	2.7	
1872.3618	1872.3603	0.8	
1873.3636	1873.3633	0.2	
1874.3608	1874.3585	1.3	
1875.3633	1875.3614	1.0	
1876.3560	1876.3567	-0.4	
1877.3589	1877.3594	-0.3	
1878.3571	1878.3550	1.1	
1879.3606	1879.3575	1.6	
1880.3542	1880.3534	0.4	
1881.3583	1881.3557	1.4	
1882.3525	1882.3519	0.3	
1883.3571	1883.3539	1.7	
1884.3519	1884.3506	0.7	
1885.3519	1885.3523	-0.2	
1886.3523	1886.3496	1.4	
1887.3529	1887.3508	1.1	
1888.3538	1888.3491	2.5	
2466.1426	2466.1455	-1.2	$[C_{21}H_{20}O_4Br_4(C_{18}H_{16}O_3Br_4)_3 + Na]^+$
2467.1550	2467.1486	2.6	
2468.1450	2468.1437	0.6	
2469.1465	2469.1466	-0.1	
2470.1423	2470.1419	0.2	
2471.1440	2471.1447	-0.3	
2472.1404	2472.1401	0.1	
2473.1428	2473.1428	0.0	
2474.1394	2474.1384	0.4	
2475.1421	2475.1409	0.5	
2476.1392	2476.1367	1.0	
2477.1423	2477.1390	1.3	
2478.1399	2478.1351	1.9	
2479.1377	2479.1371	0.2	
2480.1355	2480.1336	0.8	
2481.1338	2481.1353	-0.6	
2482.1321	2482.1321	0.0	
2483.1362	2483.1336	1.1	
2484.1294	2484.1307	-0.5	
2485.1340	2485.1319	0.8	
2486.1274	2486.1295	-0.8	
2487.1326	2487.1304	0.9	

$m/z_{measured}$	$m/z_{theoretical}$	error	assignment
3064.9189	3064.9300	-3.6	$[C_{21}H_{20}O_4Br_4(C_{18}H_{16}O_3Br_4)_4 + Na]^+$
3065.9199	3065.9253	-1.8	
3066.9214	3066.9280	-2.2	
3067.9165	3067.9236	-2.3	
3068.9246	3068.9261	-0.5	
3069.9199	3069.9219	-0.6	
3070.9219	3070.9242	-0.8	
3071.9177	3071.9202	-0.8	
3072.9199	3072.9223	-0.8	
3073.9160	3073.9185	-0.8	
3074.9187	3074.9205	-0.6	
3075.9150	3075.9169	-0.6	
3076.9182	3076.9186	-0.1	
3077.9148	3077.9153	-0.2	
3078.9119	3078.9168	-1.6	
3079.9153	3079.9138	0.5	
3080.9124	3080.9151	-0.9	
3081.9099	3081.9123	-0.8	
3082.9138	3082.9134	0.1	
3083.9114	3083.9109	0.1	
3084.9094	3084.9117	-0.7	
3085.9075	3085.9096	-0.7	
3086.9119	3086.9102	0.6	
3087.9104	3087.9084	0.7	
3088.9089	3088.9087	0.1	
3089.9075	3089.9073	0.1	
3090.9128	3090.9074	1.7	
3091.9182	3091.9064	3.8	
3092.9172	3092.9064	3.5	
3663.7224	3663.7070	4.2	$[C_{21}H_{20}O_4Br_4(C_{18}H_{16}O_3Br_4)_5 + Na]^+$
3664.6985	3664.7095	-3.0	
3665.6956	3665.7053	-2.7	
3666.6929	3666.7076	-4.0	
3667.6973	3667.7036	-1.7	
3668.6946	3668.7057	-3.0	
3669.6992	3669.7020	-0.8	
3670.6970	3670.7038	-1.9	
3671.6951	3671.7003	-1.4	
3672.7002	3672.7020	-0.5	
3673.6914	3673.6987	-2.0	
3674.6968	3674.7002	-0.9	
3675.6882	3675.6971	-2.4	
3676.6868	3676.6984	-3.2	
3677.6855	3677.6956	-2.7	
3678.6914	3678.6966	-1.4	
3679.6834	3679.6940	-2.9	
3680.6897	3680.6949	-1.4	
3681.6819	3681.6926	-2.9	
3682.6882	3682.6932	-1.3	
3683.6880	3683.6911	-0.8	
3684.6875	3684.6915	-1.1	
3685.6875	3685.6897	-0.6	
3686.6804	3686.6900	-2.6	
3687.6804	3687.6884	-2.2	
3688.6807	3688.6885	-2.1	
3689.6812	3689.6872	-1.6	
3690.6816	3690.6871	-1.5	
3691.6824	3691.6861	-1.0	

Hydration of the epoxy rings of the sodiated main series

$m/z_{measured}$	$m/z_{theoretical}$	error	assignment : + H_2O	$m/z_{theoretical}$	error	assignment : +2*H_2O
1290.5841	1290.5912	-5.5				
1292.5875	1292.5893	-1.4				
1293.5856	1293.5925	-5.4				
1294.5841	1294.5874	-2.6				
1295.5872	1295.5906	-2.6				
1296.5823	1296.5856	-2.6				
1297.5903	1297.5886	1.3				
1298.5779	1298.5840	-4.7				
1299.5825	1299.5867	-3.2				
1300.5792	1300.5825	-2.5				
1301.5887	1301.5849	2.9	$[C_{21}H_{22}O_5Br_4(C_{18}H_{16}O_3Br_4)_1 + Na]^+$			
1302.5820	1302.5814	0.5				
1303.5757	1303.5834	-5.9				
1304.5905	1304.5812	7.1				
1305.6140	1305.5825	24.1				
1306.6047	1306.5846	15.4		1306.6037	0.8	
1307.6083	1307.5870	16.2		1307.6071	0.9	
1308.5997	1308.5896	7.8		1308.6018	-1.6	
1309.6040				1309.6051	-0.8	
1310.5962				1310.5999	-2.8	$[C_{21}H_{24}O_6Br_4(C_{18}H_{16}O_3Br_4)_1 + Na]^+$
1311.6012				1311.6031	-1.5	
1312.5941				1312.5980	-3.0	
1313.5958				1313.6011	-4.0	
1314.5936				1314.5962	-2.0	
1315.6002				1315.5992	0.8	
1890.3712	1890.3708	0.2				
1891.3579	1891.3739	-8.4				
1892.3699	1892.3690	0.4				
1893.3721	1893.3719	0.1				
1894.3695	1894.3673	1.2				
1895.3672	1895.3700	-1.5				
1896.3651	1896.3656	-0.3				
1897.3684	1897.3681	0.1				
1898.3669	1898.3640	1.6				
1899.3707	1899.3663	2.3				
1900.3596	1900.3625	-1.5				
1901.3690	1901.3645	2.4				
1902.3685	1902.3612	3.8		1902.3870	-9.7	
1903.3784	1903.3629	8.2		1903.3904	-6.3	
1904.3784	1904.3602	9.6	$[C_{21}H_{22}O_5Br_4(C_{18}H_{16}O_3Br_4)_2 + Na]^+$	1904.3851	-3.5	
1905.3939	1905.3614	17.1		1905.3884	2.9	
1906.3894	1906.3597	15.6		1906.3832	3.2	
1907.3853	1907.3604	13.0		1907.3864	-0.6	
1908.3864	1908.3600	13.8		1908.3814	2.6	
1909.3828	1909.3605	11.7		1909.3845	-0.9	
1910.3844	1910.3620	11.7		1910.3796	2.5	
1911.3813	1911.3640	9.1		1911.3825	-0.6	
1912.3734	1912.3662	3.8		1912.3778	-2.3	
1913.3759				1913.3806	-2.5	$[C_{21}H_{24}O_6Br_4(C_{18}H_{16}O_3Br_4)_2 + Na]^+$
1914.3331				1914.3762	-22.5	
1915.3412				1915.3787	-19.6	
1916.3342				1916.3746	-21.1	
1917.3378				1917.3769	-20.4	
1918.3263				1918.3731	-24.4	
1919.3303				1919.3751	-23.3	
1920.3346				1920.3718	-19.4	
1921.3240				1921.3735	-25.8	
1922.3337				1922.3708	-19.3	
1923.3287				1923.3720	-22.5	
1924.3441				1924.3703	-13.6	

$m/z_{measured}$	$m/z_{theoretical}$	error	assignment : + H_2O	$m/z_{theoretical}$	error	assignment : +2*H_2O
2489.1318	2489.1553	-9.4				
2490.1318	2490.1507	-7.6				
2491.1377	2491.1533	-6.3				
2492.1438	2492.1490	-2.1				
2493.1443	2493.1514	-2.9				
2494.1509	2494.1473	1.4				
2495.1460	2495.1496	-1.4				
2496.1472	2496.1457	0.6				
2497.1484	2497.1477	0.3				
2498.1443	2498.1442	0.1		2498.1703	-10.4	
2499.1404	2499.1459	-2.2		2499.1737	-13.3	
2500.1479	2500.1427	2.1		2500.1684	-8.2	
2501.1501	2501.1442	2.4		2501.1717	-8.6	
2502.1467	2502.1413	2.2		2502.1666	-7.9	
2503.1550	2503.1425	5.0		2503.1697	-5.9	
2504.1636	2504.1401	9.4		2504.1648	-0.5	
2505.1724	2505.1410	12.5		2505.1678	1.8	
2506.1641	2506.1391	10.0	$[C_{21}H_{22}O_5Br_4(C_{18}H_{16}O_3Br_4)_3 + Na]^+$	2506.1630	0.4	
2507.1616	2507.1397	8.8		2507.1658	-1.7	
2508.1536	2508.1384	6.1		2508.1613	-3.1	
2509.1575	2509.1387	7.5		2509.1639	-2.6	
2510.1614	2510.1380	9.3		2510.1596	0.7	
2511.1597	2511.1382	8.5		2511.1620	-0.9	$[C_{21}H_{24}O_6Br_4(C_{18}H_{16}O_3Br_4)_3 + Na]^+$
2512.1467	2512.1384	3.3		2512.1579	-4.4	
2513.1340	2513.1388	-1.9		2513.1601	-10.4	
2514.1272	2514.1398	-5.0		2514.1563	-11.6	
2515.1206	2515.1414	-8.3		2515.1583	-15.0	
2516.1084				2516.1547	-18.4	
2517.1196				2517.1565	-14.7	
2518.1135				2518.1533	-15.8	
2519.1194				2519.1548	-14.0	
2520.1138				2520.1519	-15.1	
2521.1025				2521.1531	-20.1	
2522.1089				2522.1507	-16.6	
2523.1038				2523.1516	-19.0	
2524.1106				2524.1497	-15.5	
3092.9172	3092.9310	-4.5				
3093.9165	3093.9275	-3.5				
3094.9290	3094.9292	-0.1				
3095.9158	3095.9259	-3.3		3095.952	-11.6	
3096.9285	3096.9274	0.3		3096.955	-8.6	
3097.9219	3097.9244	-0.8		3097.95	-9.1	
3098.9221	3098.9257	-1.1		3098.953	-10.0	
3099.9160	3099.9229	-2.2		3099.948	-10.4	
3100.9229	3100.9239	-0.4		3100.951	-9.1	
3101.9170	3101.9215	-1.5		3101.946	-9.5	
3102.9307	3102.9223	2.7	$[C_{21}H_{22}O_5Br_4(C_{18}H_{16}O_3Br_4)_4 + Na]^+$	3102.949	-6.0	
3107.9370	3107.9179	6.1		3107.941	-1.4	
3108.9324	3108.9180	4.6		3108.943	-3.6	
3109.9341	3109.9170	5.5		3109.94	-1.8	
3110.9299	3110.9170	4.2		3110.942	-3.8	
3111.9255	3111.9164	2.9		3111.938	-4.0	
3112.9216	3112.9164	1.7		3112.94	-5.8	
3113.9114	3113.9162	-1.5		3113.936	-8.1	
3114.9075	3114.9163	-2.8		3114.938	-9.8	
3115.8975	3115.9165	-6.1		3115.935	-12.0	
3116.9006	3116.9171	-5.3		3116.936	-11.4	
3117.8909	3117.9179	-8.7		3117.933	-13.7	$[C_{21}H_{24}O_6Br_4(C_{18}H_{16}O_3Br_4)_4 + Na]^+$
3118.8813				3118.935	-17.1	
3119.8914				3119.932	-13.1	
3120.8887				3120.933	-14.2	
3121.8989				3121.931	-10.2	
3122.8899				3122.931	-13.3	
3123.8877				3123.93	-13.4	
3124.8792				3124.93	-16.2	
3125.8901				3125.929	-12.3	
3126.8818				3126.929	-15.0	
3127.9253				3127.928	-0.7	
3128.9431				3128.928	4.9	
3129.9353				3129.927	2.6	
3130.9536				3130.927	8.5	
3131.9268				3131.927	0.0	
3132.9387				3132.927	3.8	
3133.9316				3133.927	1.4	
3134.9441				3134.928	5.2	

-Br + H from the sodiated main series

$m/z_{measured}$	$m/z_{theoretical}$	error	assignment
1794.4518	1794.4498	1.1	$[C_{21}H_{21}O_4Br_3(C_{18}H_{16}O_3Br_4)_2 + Na]^+$
1795.4569	1795.4528	2.3	
1796.4478	1796.4480	-0.2	
1797.4633	1797.4509	6.9	
1798.4498	1798.4463	1.9	
1799.4513	1799.4490	1.3	
1800.4432	1800.4447	-0.8	
1801.4501	1801.4471	1.6	
1802.4425	1802.4432	-0.4	
1803.4451	1803.4453	-0.1	
1804.4430	1804.4419	0.6	
1805.4413	1805.4437	-1.3	
1806.4397	1806.4408	-0.6	
2392.2292	2392.2314	-0.9	$[C_{21}H_{21}O_4Br_3(C_{18}H_{16}O_3Br_4)_3 + Na]^+$
2393.2378	2393.2342	1.5	
2394.2295	2394.2297	-0.1	
2395.2327	2395.2323	0.2	
2396.2249	2396.2280	-1.3	
2397.2285	2397.2304	-0.8	
2398.2268	2398.2264	0.2	
2399.2310	2399.2286	1.0	
2400.2295	2400.2248	1.9	
2401.2285	2401.2267	0.7	
2402.2219	2402.2234	-0.6	
2403.2268	2403.2250	0.8	
2404.2319	2404.2220	4.1	
2405.2146	2405.2233	-3.6	
2406.2202	2406.2208	-0.2	
2990.0076	2990.0131	-1.9	$[C_{21}H_{21}O_4Br_3(C_{18}H_{16}O_3Br_4)_4 + Na]^+$
2990.9963	2991.0156	-6.5	
2992.0105	2992.0115	-0.3	
2993.0125	2993.0138	-0.4	
2994.0081	2994.0098	-0.6	
2995.0103	2995.0119	-0.5	
2996.0061	2996.0082	-0.7	
2997.0024	2997.0100	-2.5	
2998.0176	2998.0066	3.7	
2999.0078	2999.0082	-0.1	
3000.0044	3000.0051	-0.2	
3001.0012	3001.0065	-1.7	
3002.0044	3002.0036	0.3	
3002.9954	3003.0047	-3.1	
3003.9990	3004.0022	-1.1	
3005.0027	3005.0031	-0.1	
3006.0068	3006.0009	2.0	
3006.9983	3007.0015	-1.1	
3008.0088	3007.9996	3.0	
3008.9944	3009.0000	-1.9	
3591.7917	3591.7916	0.0	$[C_{21}H_{21}O_4Br_3(C_{18}H_{16}O_3Br_4)_5 + Na]^+$
3592.7649	3592.7934	-7.9	
3593.7798	3593.7900	-2.8	
3594.7813	3594.7916	-2.9	
3595.7825	3595.7884	-1.6	
3596.7771	3596.7897	-3.5	
3597.7788	3597.7868	-2.2	
3598.7805	3598.7880	-2.1	
3599.7825	3599.7853	-0.8	
3600.7776	3600.7862	-2.4	
3601.7869	3601.7838	0.8	
3602.7754	3602.7845	-2.5	
3603.7778	3603.7824	-1.3	
3604.7734	3604.7829	-2.6	
3605.7693	3605.7810	-3.2	
3606.7861	3606.7813	1.3	
3607.7546	3607.7797	-6.9	

One missing epoxy ring ($-C_3H_6O$) from the sodiated main series

$m/z_{measured}$	$m/z_{theoretical}$	error	assignment
1216.5546	1216.5544	0.1	$[C_{18}H_{16}O_3Br_4(C_{18}H_{16}O_3Br_4)_1 + Na]^+$
1217.5511	1217.5577	-5.4	
1218.5480	1218.5525	-3.7	
1219.5493	1219.5557	-5.3	
1220.5470	1220.5506	-2.9	
1221.5450	1221.5537	-7.1	
1222.5436	1222.5488	-4.2	
1223.5465	1223.5518	-4.3	
1224.5419	1224.5470	-4.2	
1225.5415	1225.5499	-6.8	
1226.5377	1226.5454	-6.3	
1227.5463	1227.5480	-1.4	
1228.5432	1228.5442	-0.8	
1816.3367	1816.3340	1.5	$[C_{18}H_{16}O_3Br_4(C_{18}H_{16}O_3Br_4)_2 + Na]^+$
N.D.	1817.3371	N.D.	
1818.3350	1818.3322	1.5	
1819.3273	1819.3351	-4.3	
1820.3345	1820.3304	2.2	
1821.3370	1821.3332	2.1	
1822.3301	1822.3287	0.8	
1823.3284	1823.3313	-1.6	
1824.3268	1824.3270	-0.1	
1825.3256	1825.3294	-2.1	
1826.3246	1826.3255	-0.5	
1827.3289	1827.3276	0.7	
2416.1118	2416.1138	-0.8	$[C_{18}H_{16}O_3Br_4(C_{18}H_{16}O_3Br_4)_3 + Na]^+$
2417.1140	2417.1165	-1.0	
2418.1106	2418.1121	-0.6	
2419.1189	2419.1146	1.8	
2420.1104	2420.1104	0.0	
2421.1133	2421.1127	0.2	
2422.1108	2422.1088	0.9	
2423.1143	2423.1109	1.4	
2424.1064	2424.1072	-0.3	
2425.1104	2425.1090	0.5	
2426.1030	2426.1057	-1.1	
2427.1072	2427.1073	0.0	
2428.1060	2428.1043	0.7	
2429.1106	2429.1056	2.1	
2430.1099	2430.1030	2.8	
3015.8848	3015.8938	-3.0	$[C_{18}H_{16}O_3Br_4(C_{18}H_{16}O_3Br_4)_4 + Na]^+$
N.D.	3016.8961	N.D.	
3017.8901	3017.8922	-0.7	
3018.8962	3018.8942	0.7	
3019.8835	3019.8905	-2.3	
3020.8901	3020.8923	-0.7	
3021.8904	3021.8889	0.5	
3022.8909	3022.8905	0.1	
3023.8977	3023.8874	3.4	
3024.8860	3024.8887	-0.9	
3025.8740	3025.8859	-3.9	
3026.8816	3026.8870	-1.8	
3027.9019	3027.8845	5.7	
3028.8779	3028.8854	-2.5	
3615.6731	3615.6740	-0.2	$[C_{18}H_{16}O_3Br_4(C_{18}H_{16}O_3Br_4)_5 + Na]^+$
N.D.	3616.6757	N.D.	
3617.6680	3617.6724	-1.2	
N.D.	3618.6739	N.D.	
3619.6704	3619.6708	-0.1	
3620.6682	3620.6721	-1.1	
3621.6523	3621.6692	-4.6	
3622.6714	3622.6703	0.3	
3623.6558	3623.6676	-3.3	
3624.6543	3624.6685	-3.9	
3625.6667	3625.6661	0.2	
N.D.	3626.6668	N.D.	
3627.6714	3627.6647	1.8	
3628.6494	3628.6652	-4.3	

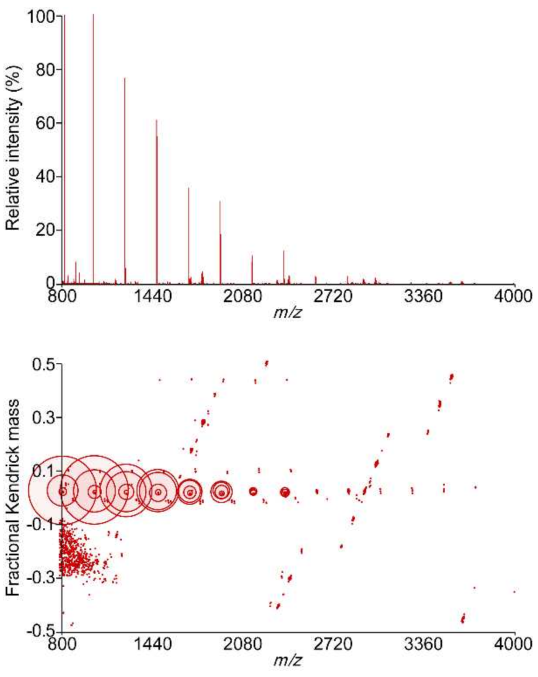

Figure S7. MALDI-spiralTOF mass spectrum of heated PBT / brominated flame retardant / Sb_2O_3 (T=330 degC, 5 min). Bottom: Kendrick plot using R=220.0763 and x=220.

Tables S4. Accurate mass measurements and assignments for the loss of bromine (-Br + H or -HBr) from the main series detected in the PBT / flame retardant / Sb2O3 sample after heating. Errors are expressed in ppm. PBT peaks are omitted (same series as listed in the previous table). Peaks are selected from the Kendrick plots using kendo (polygon selection / export). Errors are indifferently computed using Excel from the peak list or kendo from the Kendrick plot (simulation + polygon selection / compute / error / plot / export).

$m/z_{measured}$	$m/z_{theoretical}$	error	assignment : -H + Br	$m/z_{theoretical}$	error	assignment : -HBr
1192.6464	1192.672	-21.5	$[C_{21}H_{21}O_4Br_3(C_{18}H_{16}O_3Br_4)_1 + Na]^+$ = - Br+ H	1192.6545	-6.8	$[C_{21}H_{19}O_4Br_3(C_{18}H_{16}O_3Br_4)_1 + Na]^+$ = - HBr
1194.6478	1194.67	-18.7		1194.6526	-4.0	
1195.6592	1195.673	-11.9		1195.6558	2.8	
1196.6510	1196.668	-14.4		1196.6508	0.2	
1197.6472	1197.671	-20.2		1197.6539	-5.5	
1198.6479	1198.666	-15.5		1198.6491	-1.0	
1199.6529	1199.67	-13.9		1199.6520	0.8	
1200.6464	1200.665	-15.3		1200.6475	-0.9	
1201.6443	1201.668	-19.4		1201.6501	-4.9	
1202.6506	1202.663	-10.5		1202.6463	3.5	
1203.6493	1203.666	-13.7		1203.6485	0.7	
1272.5802	1272.5806	-0.4	$[C_{21}H_{20}O_4Br_4(C_{18}H_{16}O_3Br_4)_1 + Na]^+$ = main series			
1274.5777	1274.5787	-0.8				
1275.5812	1275.5820	-0.6				
1276.5768	1276.5769	0.0				
1277.5770	1277.5800	-2.4				
1278.5734	1278.5751	-1.3				
1279.5743	1279.5780	-2.9				
1280.5715	1280.5734	-1.5				
1281.5732	1281.5762	-2.3				
1282.5712	1282.5719	-0.6				
1283.5736	1283.5744	-0.6				
1284.5641	1284.5707	-5.2				
1714.5080	1714.5411	-19.3	$[C_{21}H_{22}O_4Br_2(C_{18}H_{16}O_3Br_4)_2 + Na]^+$ = - 2Br + 2H	1714.5063	1.0	$[C_{21}H_{18}O_4Br_2(C_{18}H_{16}O_3Br_4)_2 + Na]^+$ = - 2HBr
1716.5068	1716.5394	-19.0		1716.5047	1.2	
1718.5067	1718.5376	-18.0		1718.5032	2.0	
1720.5030	1720.5360	-19.2		1720.5018	0.7	
1790.4407	1790.4535	-7.1	$[C_{21}H_{21}O_4Br_3(C_{18}H_{16}O_3Br_4)_2 + Na]^+$ = - Br+ H	1790.4360	2.7	$[C_{21}H_{19}O_4Br_3(C_{18}H_{16}O_3Br_4)_2 + Na]^+$ = - HBr
1792.4345	1792.4516	-9.5		1792.4341	0.2	
1793.4342	1793.4548	-11.5		1793.4372	-1.6	
1794.4342	1794.4498	-8.7		1794.4324	1.0	
1795.4394	1795.4528	-7.5		1795.4352	2.3	
1796.4302	1796.4480	-9.9		1796.4307	-0.3	
1797.4360	1797.4509	-8.3		1797.4333	1.5	
1798.4322	1798.4463	-7.9		1798.4291	1.7	
1799.4336	1799.4490	-8.6		1799.4315	1.2	
1800.4304	1800.4447	-7.9		1800.4276	1.6	
1801.4324	1801.4471	-8.2		1801.4297	1.5	
1802.4297	1802.4432	-7.5		1802.4262	1.9	
1803.4322	1803.4453	-7.3		1803.4280	2.3	
1804.4301	1804.4419	-6.5		1804.4252	2.7	
1870.3590	1870.3621	-1.6	$[C_{21}H_{20}O_4Br_4(C_{18}H_{16}O_3Br_4)_2 + Na]^+$ = main series			
1872.3619	1872.3603	0.9				
1873.3638	1873.3633	0.2				
1874.3609	1874.3585	1.3				
1875.3633	1875.3614	1.0				
1876.3559	1876.3567	-0.4				
1877.3588	1877.3594	-0.3				
1878.3570	1878.3550	1.1				
1879.3555	1879.3575	-1.1				
1880.3542	1880.3534	0.4				
1881.3581	1881.3557	1.3				
1882.3524	1882.3519	0.2				
1883.3519	1883.3539	-1.1				
1884.3517	1884.3506	0.6				
1885.3517	1885.3523	-0.3				
1886.3520	1886.3496	1.3				
1887.3526	1887.3508	0.9				

$m/z_{measured}$	$m/z_{theoretical}$	error	assignment : -H + Br	$m/z_{theoretical}$	error	assignment : -HBr
2312.2848	2312.3227	-16.4	$[C_{21}H_{22}O_4Br_2(C_{18}H_{16}O_3Br_4)_3 + Na]^+$ = - 2Br + 2H	2312.2880	-1.4	$[C_{21}H_{18}O_4Br_2(C_{18}H_{16}O_3Br_4)_3 + Na]^+$ = - 2HBr
2313.2929	2313.3257	-14.2		2313.2905	1.0	
2314.2846	2314.3210	-15.7		2314.2864	-0.8	
2315.2875	2315.3237	-15.6		2315.2887	-0.5	
2316.2796	2316.3193	-17.1		2316.2848	-2.2	
2317.2885	2317.3218	-14.4		2317.2869	0.7	
2318.2866	2318.3177	-13.4		2318.2833	1.4	
2319.2904	2319.3200	-12.7		2319.2851	2.3	
2320.2834	2320.3161	-14.1		2320.2819	0.6	
2321.2877	2321.3182	-13.1		2321.2834	1.8	
2322.2810	2322.3146	-14.5		2322.2807	0.1	
2390.2166	2390.2332	-6.9	$[C_{21}H_{21}O_4Br_3(C_{18}H_{16}O_3Br_4)_3 + Na]^+$ = - Br+ H	2390.2158	0.3	$[C_{21}H_{19}O_4Br_3(C_{18}H_{16}O_3Br_4)_3 + Na]^+$ = - HBr
2391.2134	2391.2361	-9.5		2391.2186	-2.2	
2392.2160	2392.2314	-6.5		2392.2141	0.8	
2393.2131	2393.2342	-8.8		2393.2167	-1.5	
2394.2105	2394.2297	-8.0		2394.2124	-0.8	
2395.2137	2395.2323	-7.8		2395.2148	-0.4	
2396.2115	2396.2280	-6.9		2396.2108	0.3	
2397.2152	2397.2304	-6.4		2397.2129	0.9	
2398.2077	2398.2264	-7.8		2398.2092	-0.6	
2399.2118	2399.2286	-7.0		2399.2111	0.3	
2400.2104	2400.2248	-6.0		2400.2077	1.1	
2401.2092	2401.2267	-7.3		2401.2093	0.0	
2402.2083	2402.2234	-6.3		2402.2063	0.8	
2403.2075	2403.2250	-7.3		2403.2077	0.0	
2404.2070	2404.2220	-6.2		2404.2051	0.8	
2405.2067	2405.2233	-6.9		2405.2061	0.2	
2406.2122	2406.2208	-3.6		2406.2041	3.4	
2470.1441	2470.1419	0.9	$[C_{21}H_{20}O_4Br_4(C_{18}H_{16}O_3Br_4)_3 + Na]^+$ = main series			
2471.1459	2471.1447	0.5				
2472.1365	2472.1401	-1.5				
2473.1387	2473.1428	-1.6				
2474.1354	2474.1384	-1.2				
2475.1381	2475.1409	-1.1				
2476.1352	2476.1367	-0.6				
2477.1382	2477.1390	-0.3				
2478.1357	2478.1351	0.2				
2479.1392	2479.1371	0.8				
2480.1371	2480.1336	1.4				
2481.1352	2481.1353	-0.1				
2482.1335	2482.1321	0.6				
2483.1378	2483.1336	1.7				
2484.1308	2484.1307	0.0				
2485.1297	2485.1319	-0.9				
2486.1288	2486.1295	-0.3				

$m/z_{measured}$	$m/z_{theoretical}$	error	assignment : -H + Br	$m/z_{theoretical}$	error	assignment : -HBr
2834.1406	2834.1923	-18.3	$[C_{21}H_{23}O_4Br(C_{18}H_{16}O_3Br_4)_4 + Na]^+$ = - 3Br + 3H	2834.1406	0.0	$[C_{21}H_{17}O_4Br(C_{18}H_{16}O_3Br_4)_4 + Na]^+$ = - 3HBr
2836.1336	2836.1907	-20.1		2836.1391	-1.9	
2837.1335	2837.1928	-20.9		2837.1405	-2.5	
2838.1397	2838.1891	-17.4		2838.1377	0.7	
2910.0652	2910.1044	-13.5	$[C_{21}H_{22}O_4Br_2(C_{18}H_{16}O_3Br_4)_4 + Na]^+$ = - 2Br + 2H	2910.0698	-1.6	$[C_{21}H_{18}O_4Br_2(C_{18}H_{16}O_3Br_4)_4 + Na]^+$ = - 2HBr
2911.0780	2911.1071	-10.0		2911.0720	2.1	
2912.0662	2912.1027	-12.6		2912.0681	-0.7	
2913.0669	2913.1052	-13.1		2913.0701	-1.1	
2914.0616	2914.1011	-13.5		2914.0665	-1.7	
2915.0689	2915.1033	-11.8		2915.0683	0.2	
2916.0639	2916.0994	-12.2		2916.0650	-0.4	
2917.0653	2917.1014	-12.4		2917.0665	-0.4	
2918.0607	2918.0979	-12.7		2918.0635	-1.0	
2919.0562	2919.0996	-14.9		2919.0648	-2.9	
2920.0644	2920.0963	-10.9		2920.0621	0.8	
2921.0602	2921.0978	-12.9		2921.0631	-1.0	
2922.0625	2922.0948	-11.1		2922.0608	0.6	
2923.0649	2923.0961	-10.7		2923.0615	1.2	
2924.0613	2924.0934	-11.0		2924.0596	0.6	
2989.9842	2990.0131	-9.7	$[C_{21}H_{21}O_4Br_3(C_{18}H_{16}O_3Br_4)_4 + Na]^+$ = - Br + H	2989.9958	-3.9	$[C_{21}H_{19}O_4Br_3(C_{18}H_{16}O_3Br_4)_4 + Na]^+$ = - HBr
2990.9919	2991.0156	-7.9		2990.9981	-2.1	
2991.9935	2992.0115	-6.0		2991.9941	-0.2	
2992.9953	2993.0138	-6.2		2992.9962	-0.3	
2993.9846	2994.0098	-8.4		2993.9925	-2.6	
2994.9930	2995.0119	-6.3		2994.9944	-0.5	
2995.9890	2996.0082	-6.4		2995.9909	-0.7	
2996.9914	2997.0100	-6.2		2996.9926	-0.4	
2997.9877	2998.0066	-6.3		2997.9894	-0.6	
2998.9842	2999.0082	-8.0		2998.9908	-2.2	
2999.9808	3000.0051	-8.1		2999.9879	-2.4	
3000.9839	3001.0065	-7.5		3000.9891	-1.7	
3001.9872	3002.0036	-5.5		3001.9865	0.2	
3002.9843	3003.0047	-6.8		3002.9874	-1.0	
3003.9879	3004.0022	-4.8		3003.9852	0.9	
3004.9790	3005.0031	-8.0		3004.9859	-2.3	
3005.9767	3006.0009	-8.1		3005.9840	-2.4	
3069.9136	3069.9219	-2.7	$[C_{21}H_{20}O_4Br_4(C_{18}H_{16}O_3Br_4)_4 + Na]^+$ = main series			
3070.9220	3070.9242	-0.7				
3071.9177	3071.9202	-0.8				
3072.9200	3072.9223	-0.8				
3073.9097	3073.9185	-2.9				
3074.9187	3074.9205	-0.6				
3075.9151	3075.9169	-0.6				
3076.9116	3076.9186	-2.3				
3077.9147	3077.9153	-0.2				
3078.9116	3078.9168	-1.7				
3079.9086	3079.9138	-1.7				
3080.9122	3080.9151	-0.9				
3081.9096	3081.9123	-0.9				
3082.9071	3082.9134	-2.0				
3083.9048	3083.9109	-2.0				
3084.9154	3084.9117	1.2				
3085.9070	3085.9096	-0.8				
3086.9052	3086.9102	-1.6				
3087.9035	3087.9084	-1.6				

*m/z*measured	*m/z*theoretical	error	assignment -H + Br	*m/z*theoretical	error	assignment -HBr
3509.8427	3509.8845	-11.9		3509.8499	-2.1	
3511.8423	3511.8828	-11.6		3511.8483	-1.7	
3512.8252	3512.8848	-17.0		3512.8498	-7.0	
3513.8492	3513.8812	-9.1		3513.8468	0.7	
3514.8460	3514.8829	-10.5		3514.8480	-0.6	
3515.8362	3515.8796	-12.4		3515.8453	-2.6	
3516.8470	3516.8811	-9.7	$[C_{21}H_{22}O_4Br_2(C_{18}H_{16}O_3Br_4)_5 + Na]^+$ = - 2Br + 2H	3516.8463	0.2	$[C_{21}H_{18}O_4Br_2(C_{18}H_{16}O_3Br_4)_5 + Na]^+$ = - 2HBr
3517.8306	3517.8781	-13.5		3517.8438	-3.7	
3518.8485	3518.8794	-8.8		3518.8446	1.1	
3519.8324	3519.8766	-12.6		3519.8423	-2.8	
3520.8369	3520.8776	-11.6		3520.8429	-1.7	
3521.8416	3521.8751	-9.5		3521.8409	0.2	
3522.8327	3522.8759	-12.3		3522.8414	-2.4	
3589.7710	3589.7932	-6.2		3589.7759	-1.4	
3590.7855	3590.7952	-2.7		3590.7777	2.2	
3591.7725	3591.7916	-5.3		3591.7743	-0.5	
3592.7596	3592.7934	-9.4		3592.7759	-4.5	
3593.7676	3593.7900	-6.2		3593.7727	-1.4	
3594.7619	3594.7916	-8.2		3594.7741	-3.4	
3595.7702	3595.7884	-5.1		3595.7712	-0.3	
3596.7716	3596.7897	-5.0	$[C_{21}H_{21}O_4Br_3(C_{18}H_{16}O_3Br_4)_5 + Na]^+$ = - Br + H	3596.7723	-0.2	$[C_{21}H_{19}O_4Br_3(C_{18}H_{16}O_3Br_4)_5 + Na]^+$ = - HBr
3597.7595	3597.7868	-7.6		3597.7697	-2.8	
3598.7612	3598.7880	-7.4		3598.7706	-2.6	
3599.7631	3599.7853	-6.2		3599.7682	-1.4	
3600.7721	3600.7862	-3.9		3600.7689	0.9	
3601.7605	3601.7838	-6.5		3601.7667	-1.7	
3602.7628	3602.7845	-6.0		3602.7672	-1.2	
3603.7583	3603.7824	-6.7		3603.7653	-1.9	
3604.7679	3604.7829	-4.2		3604.7657	0.6	
3669.6922	3669.7020	-2.7				
3670.6899	3670.7038	-3.8				
3671.6879	3671.7003	-3.4				
3672.6929	3672.7020	-2.5				
3673.6981	3673.6987	-0.2				
3674.6894	3674.7002	-2.9				
3675.6879	3675.6971	-2.5				
3676.7004	3676.6984	0.6				
3677.6852	3677.6956	-2.8	$[C_{21}H_{20}O_4Br_4(C_{18}H_{16}O_3Br_4)_5 + Na]^+$ = main series			
3678.6980	3678.6966	0.4				
3679.6830	3679.6940	-3.0				
3680.6822	3680.6949	-3.4				
3681.6815	3681.6926	-3.0				
3682.6879	3682.6932	-1.4				
3683.6804	3683.6911	-2.9				
3684.6801	3684.6915	-3.1				
3685.6869	3685.6897	-0.8				
3686.6729	3686.6900	-4.6				
